# Preserving *π*-conjugation in covalently functionalized carbon nanotubes for optoelectronic applications

**DOI:** 10.1038/ncomms14281

**Published:** 2017-01-30

**Authors:** Antonio Setaro, Mohsen Adeli, Mareen Glaeske, Daniel Przyrembel, Timo Bisswanger, Georgy Gordeev, Federica Maschietto, Abbas Faghani, Beate Paulus, Martin Weinelt, Raul Arenal, Rainer Haag, Stephanie Reich

**Affiliations:** 1 Department of Physics, Free University Berlin, Arnimallee 14, 14195 Berlin, Germany; 2 Faculty of Science, Department of Chemistry, Lorestan University, Khorram Abad 68151-44316, Iran; 3 Institute of Chemistry and Biochemistry, Free University Berlin, 14195 Berlin, Germany; 4 Laboratorio de Microscopias Avanzadas (LMA), Instituto de Nanociencia de Aragon, Universidad de Zaragoza, 50018 Zaragoza, Spain; 5 Fundacion ARAID, 50018 Zaragoza, Spain

## Abstract

Covalent functionalization tailors carbon nanotubes for a wide range of applications in varying environments. Its strength and stability of attachment come at the price of degrading the carbon nanotubes *sp*^2^ network and destroying the tubes electronic and optoelectronic features. Here we present a non-destructive, covalent, gram-scale functionalization of single-walled carbon nanotubes by a new [2+1] cycloaddition. The reaction rebuilds the extended *π*-network, thereby retaining the outstanding quantum optoelectronic properties of carbon nanotubes, including bright light emission at high degree of functionalization (1 group per 25 carbon atoms). The conjugation method described here opens the way for advanced tailoring nanotubes as demonstrated for light-triggered reversible doping through photochromic molecular switches and nanoplasmonic gold-nanotube hybrids with enhanced infrared light emission.

## Abstract (web summary)

While covalent modification of carbon nanotubes allows their use in a wide range of applications, it often results in disruption of their optoelectronic properties. Here, the authors design a cycloaddition reaction that preserves the nanotubes electronic structure.

The unique optoelectronic properties of carbon nanotubes originate from their singular mixture of *sp*^2^ carbon bonding, one-dimensional character and suppressed dielectric screening^1^. This makes them ideal building blocks for applied optoelectronics as nanometre-scale light sources, photodetectors and photovoltaic devices^2^. Nanotubes were demonstrated as optical rectifying antennas converting electromagnetic radiation at optical frequencies to direct current^3^ and as single-photon sources at room temperature^4^. Their stability and compatibility with many environments, including biological systems, makes them optically detectable carriers of drugs and radiotracers as demonstrated for *in vivo* localization and imaging^5^. Bio-imaging greatly benefits from the wavelength at which single-walled carbon nanotubes (SWNTs) emit light; it lies in the second window of tissue transparency (1,100–1,400 nm) with a large penetration depth, but low tissue scattering and autofluorescence^6^,^7^.

Optoelectronic applications heavily rely on the read-out of the SWNT fluorescence. For this, the *π*-conjugated structure of the individual nanotube needs to be preserved^8^, as optical excitation and emission of SWNTs are ruled by the *π*-electrons of the carbon backbone^1^,^8^,^9^. A disruption of the *π*-network by rehybridization from the *sp*^2^ to the *sp*^3^ configuration degrades the conjugation, increases the number of non-radiative scattering centres and quenches the overall SWNTs luminescence^10^. At the same time, the *π*-conjugation and *π*–*π* interactions are the driving force of a strong tube bundling. This prevents luminescence through energy transfer into metallic tubes followed by non-radiative recombination^8^.

Only individual SWNTs are strong light emitters and various types of functionalization have been pursued to isolate, stabilize and tailor them^11^. Endohedral functionalization fills the nanotubes, whereas covalent and non-covalent exohedral functionalizations operate onto the SWNT sidewalls^11^. Endohedral filling preserved the electronic properties of SWNTs^12^,^13^ but is limited to small molecules that fit into the tubes (inner diameter <2 nm). The exohedral non-covalent approach is based on physisorption of the targeted functionality onto the SWNTs^11^. It preserves the *π*-conjugation of the *sp*^2^ network. However, changes in the environment easily reverse the functionalization and trigger desorption making this method inherently unstable. The covalent exohedral approach is advantageous in that it strongly binds the functionalities onto the SWNTs by converting a fraction of the *sp*^2^ into *sp*^3^ carbons. Its current implementations, however, interrupt the electronic properties of the nanotubes and quench their luminescence^8^,^10^,^11^,^14^. Alternative approaches such as mechanically interlocking nanotubes likewise resulted in reduced light emission^15^.

New covalent methods are needed, which combine the *π*-conjugation of the SWNTs with a stable functionalization. They will have to operate under mild conditions and rebuild a fully *sp*^2^ *π*-conjugated system. The [2+1] cycloaddition reactions have the potential to solve this challenge, because they exploit the *π* electrons for attachment instead of requiring dangling bonds^16^,^17^,^18^. A first example of such mild [2+1] cycloaddition has been recently reported based on an electron-poor aromatic azide^19^,^20^. This reaction initially transforms two *π*-electrons of the carbon network to a covalent three-membered ring bridge (closed configuration). A subsequent rehybridization step that releases the strained C–C bond underlying the bridge and reconverts the two C atoms back to the *sp*^2^ state recovering the aromaticity of the system (open configuration) has only been studied theoretically^21^,^22^,^23^. Lee and Marzari^21^ proposed to exploit the open configuration with dichlorocarbene to not disrupt the transport properties of metallic SWNTs. Experimentally, however, neither the carbene nor the nitrene addition have yielded the open structure and no improvement of the optoelectronic properties of the nanotubes has been reported^16^,^18^,^24^,^25^.

Here we develop a [2+1] cycloaddition reaction based on electron-poor aromatic nitrenes that preserves the *π*-conjugated electronic structure and infrared light emission. Our functionalization method is universal and may conjugate many different functionalities to the surface of SWNTs. It is highly robust, but non-destructive for the unique properties of carbon nanotubes. The functional groups become an integral part of the extended conjugated network. We show light-triggered reversible doping of SWNTs for nanotubes with photochromic molecular switches. Gold nanoparticles (AuNPs) offer a covalent attachment of plasmonic structures, leading to an enhanced luminescence. The two examples highlight the versatility of our platform for hybrid systems with distinctive photoelectronic properties.

## Results

### *π*-Preserving triazine conjugation onto the SWNTs

**Figure 1:**
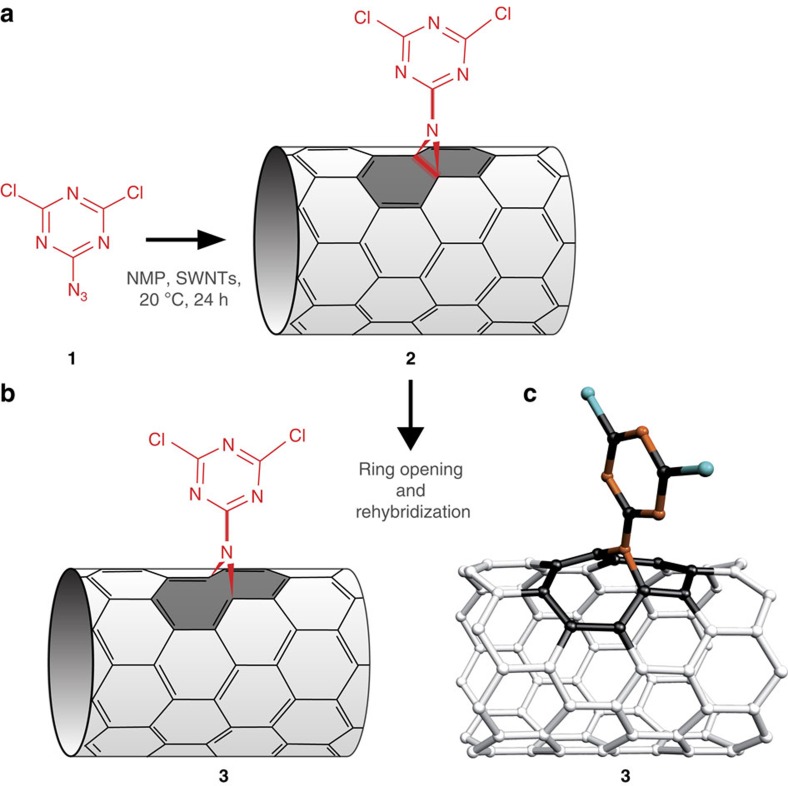
One-pot functionalization of carbon nanotubes by heterocyclic [2+1] cycloaddition reaction. (**a**) After establishment of the heterocyclic bridge between azidodichloro-triazine **1** and SWNTs, (**b**) the cycloaddition product (**2**) undergoes ring opening and rehybridization of the C atoms underlying the bridge and is converted into the form (**3**) with regenerated *π*-conjugation. (**c**) Quantum chemically optimized molecular configuration of the triazine on an (8,0) nanotube.

We introduce a [2+1] reaction based on azidodichloro-triazine **1** (Fig. 1) that *in situ* generates the corresponding nitrene to form the intermediate SWNT adduct (**2**). As predicted for such highly strained intermediates, ring-opening and rehybridization forms fully conjugated hetero-bridged nanotubes (**3**) in a single synthetic step. In this way, the electron lone pair of the bridging nitrogen atom becomes part of the *π*-conjugated system of the SWNT, increasing its electron density.

The highly reactive electron-poor precursor monoazidodichloro-triazine **1** was selectively prepared at 0 °C *in situ* from commercially available compounds^26^,^27^ (Supplementary Fig. 1 and Supplementary Note 1). It conjugates onto SWNT through [2+1] cycloaddition at 20 °C to form intermediate **2**, which ring opens to the final rehybridized structure **3**. Quantum chemical calculations predict that the entire reaction proceeds without activation barrier at room temperature (300 K; Supplementary Fig. 17). In the fully relaxed structure (Fig. 1c), the triazine covalently bridges onto the nanotubes with a binding energy of 4.2 eV. The covalent attachment was also proven by thermogravimetric analysis (Supplementary Fig. 5). The obtained open configuration minimally distorts the carbon *π*-orbitals (Supplementary Note 2). The number of functional groups attached to the nanotubes is adjusted by the reaction temperature (20–70 °C)^28^,^29^. We present exemplary a low-density functionalization with one triazine ring per 100 carbon atoms (SWNT-low) and a high-density functionalization with one triazine ring per 25 carbon atoms (SWNT-high). For details on the synthesis, the characterization of the intermediate products and the functionalized SWNTs, please refer to the Method section and Supplementary Notes 1 and 3.

**Figure 2:**
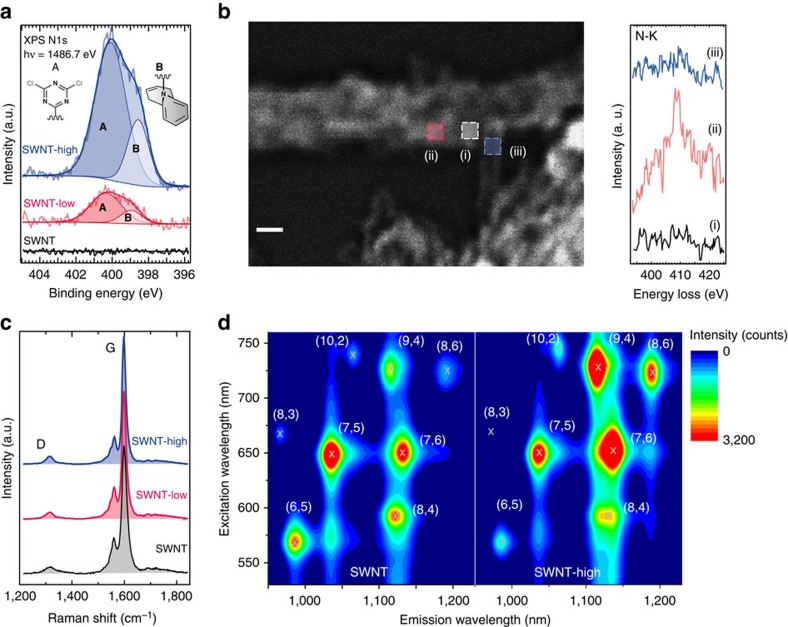
Demonstration of *π*-conjugation preserving functionalization. (**a**) X-ray photoelectron spectroscopy (XPS) spectra of the N1s level of pristine and functionalized SWNTs. (**b**) High-resolution STEM–high-angle annular dark field (HRSTEM–HAADF) micrograph of the SWNT-high sample, showing a bundle of SWNTs and an individual tube. Scale bar, 2 nm. The regions marked by squares were investigated by EELS, see Supplementary Information. The inset on the right shows the corresponding N-K edge EEL spectra. (**c**) Raman spectra showing the D and G bands of samples with different density of functional groups. (**d**) Two-dimensional (2D) luminescence maps of pristine SWNTs compared with SWNT-high. The (*n*,*m*) indices specify the SWNT species associated with each emission spot.

X-ray photoelectron spectroscopy of the N1s level shows that the pristine SWNT sample was free of nitrogen (Fig. 2a). The triazine conjugated samples **3** (SWNT-low and SWNT-high) clearly display nitrogen peaks (Fig. 2a), which increased by a factor of 4.3±0.5 between the two samples, in agreement with the elemental analysis (see Supplementary Note 3). This proves the successful tuneable decoration of the nanotubes with dichlorotriazine. With increasing nitrogen coverage we observe an exponential and uniform shift of the C1s XP spectra to higher binding energy (up to 200 meV for SWNT-high, see Supplementary Fig. 29). This reflects an increasing Fermi energy, because the nitrogen lone pair interacts with the nanotube. High-resolution scanning transmission electron microscopy (STEM) and electron energy loss spectroscopy (EELS) prove the outcome of the functionalization on a single nanotube level^30^. In Fig. 2b, we show a high-angle annular dark field high-resolution STEM micrograph of a SWNT-high together with spatially resolved EELS spectra at the nitrogen edge. The spectra, taken in the regions marked by squares in the STEM image, clearly prove the localization of nitrogen in the external sidewall of the SWNTs (region ii, Fig. 2b right).

Our covalent functionalization is unique as it preserves the *sp*^2^ character of the conjugated carbon nanotubes, see EELS analyses in Supplementary Note 1. Raman spectra show a constant intensity of the defect-induced D-mode (Fig. 2c)^9^. The ratio between the D and G bands reflects the fraction of *sp*^3^ atoms and other defects in an *sp*^2^ carbon system. Even at highest functionalization (SWNT-high, one triazine ring per 25 carbon atoms) the ratio remained identical to the pristine material *I*_D_/*I*_G_=0.1, proving that no conversion of C atoms from *sp*^2^ to *sp*^3^ occurred. The outcome of conjugating triazine (**1**) onto the SWNT is thus the ring-open compound (**3**).

The triazine-functionalized SWNTs show strong light emission, because the *π*-network was kept intact. The overall two-dimensional luminescence intensity of SWNT-high is identical to that of the pristine tubes (Fig. 2d). Each of the spots in Fig. 2d univocally confirms the presence of a specific (*n,m*) SWNT species^8^ and their unperturbed *π*-conjugated system for the SWNT-high sample^10^. Some functionalized species show even brighter emission than their pristine counterparts (compare, for example, the (9,4) tube in the left and right panel of Fig. 2d). The increase in light emission is due to the change in chemical potential by the triazine conjugated onto the tubes. Raman measurements showed an initial position of the Fermi level 70 meV away from the Dirac point (see Supplementary Fig. 30, Supplementary Table 6 and Supplementary Note 4). For SWNT-high, the Fermi level is within 20 meV of the intrinsic value, which increases the luminescence intensity. Emission from highly covalently functionalized carbon nanotubes has never been reported before and disproves the dogma that covalent functionalization always quenches nanotube emission.

Advanced photoelectronic applications of SWNTs require further customization of the tubes. For optoelectronics, for example, the emission should be controlled through external parameters. Sensing and imaging in biological environment, on the other hand, benefit from enhanced overall emission. With such applications in mind, we present two functional examples for covalently tailored luminescent SWNTs: A conjugated photochromic molecular switch to control the emission from the SWNTs and covalently attached AuNPs to plasmonically enhance the nanotubes optical response.

### Photochromic molecular switches-based conjugation

**Figure 3:**
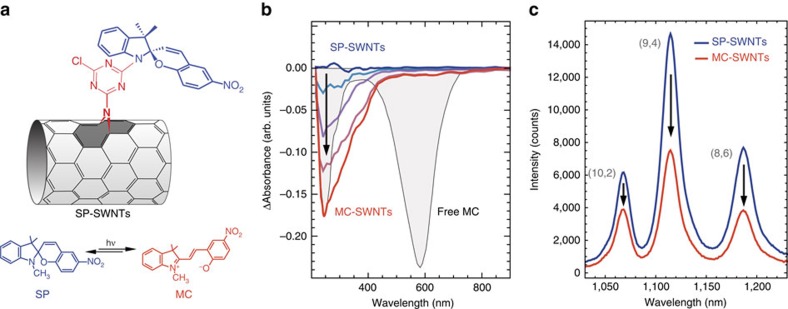
SP-conjugated SWNTs. (**a**) Schematic representation of the SP-SWNTs. (**b**) Absorption spectrum of the SP-SWNTs under ultraviolet light irradiation at 367 nm. Grey: absorption spectrum of free MC. (**c**) Emission spectra of the SP-SWNTs (blue) and of the MC-SWNTs (red), obtained after ultraviolet irradiation.

Photochromic molecular switches are molecular systems displaying two or more (meta-)stable configurations with distinctive chemo-physical features^31^. On irradiation with photons, they change their configuration and properties. As a prominent example, spiropyran (SP) on irradiation with ultraviolet light (*λ*=350 nm) passes from the closed to the open merocyanine (MC) form (see Fig. 3). We covalently connected SP to SWNTs, which made the switch an active part of the extended *π*-network (Fig. 3a, see Methods and Supplementary Note 1 for the synthesis and characterization).

The SWNTs carried one SP group every 100 carbon atoms. Under ultraviolet irradiation the SP-functionalized SWNTs (SP-SWNTs) hybrids convert into MC-SWNTs by the molecular switching from SP to MC as seen by the characteristic change in the ultraviolet absorption band in Fig. 3b and Supplementary Fig. 33. The isomerization is reversible by keeping the sample in darkness for 24 h. Interestingly, the light-induced back isomerization, MC-to-SP, is supressed for functionalized SWNTs. Indeed, MC-SWNTs display no characteristic band in the visible reminiscent of the free MC (Fig. 3b). This absorption is observed for free MC and ascribed to the *π*-electron delocalized along the MC structure^32^. For MC-SWNT, the *π*-electron is no longer confined to the MC but can extend over the whole conjugated MC-SWNTs network. This delocalization over a few hundreds of nanometres shifts the transition energy in the far infrared outside of our measurement windows^33^. The net effect of the SP to MC isomerization on the SWNTs is a further increase in the Fermi level. We effectively and reversibly dope the SWNTs by exposing them to ultraviolet photons. This idea is supported by a shift of the Raman G-band from SP-SWNTs to MC-SWNTs (see Supplementary Fig. 32)^34^.

The shift of 2 cm^−1^ in semiconducting tubes corresponds to 0.2 eV change in the energy of the Fermi level^34^. This also reduced the rate of radiative exciton emission^35^,^36^,^37^. We observed a quenching of 50% emission intensity when passing from SP- to the MC-SWNTs (see Fig. 3c). The intensity fully recovers when MC thermally isomerizes back to SP.

Covalently functionalizing SWNTs with SP thus offers a novel controlled, non-disruptive and reversible way to tailor the optoelectronic properties of nanotubes. Through ultraviolet photons we change the Fermi level and modulate light emission by SWNTs. SP-SWNTs could lead to light-switchable ballistic transport channels.

### Plasmonically enhanced emission

**Figure 4:**
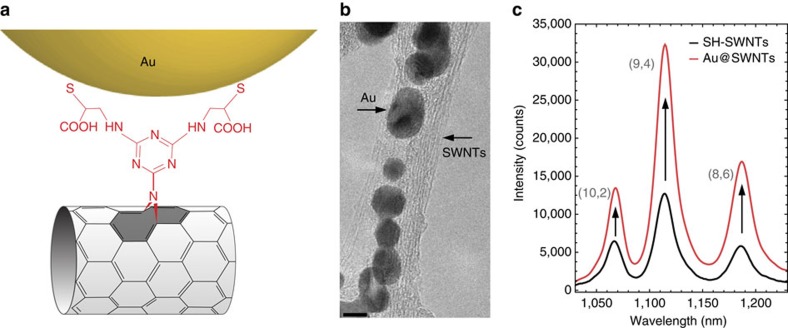
Plasmonic hybrids of AuNP and SWNT. (**a**) Molecular sketch of AuNPs covalently anchored to SH-SWNTs. (**b**) TEM micrograph of Au@SWNTs hybrids. Scale bar, 5 nm. A few-SWNTs bundle can be observed and AuNPs assembled along the tubes. (**c**) Enhancement of the luminescence emission of SWNTs after covalent attachment of AuNPs onto their surface: comparison of the emission of the Au@SWNT hybrids (red curve) with the one of SH-SWNT (black curve).

In a second application we demonstrate nanoplasmonic-hybridized SWNTs as brighter fluorophores, for example, for increasing resolution in bio-imaging^6^,^7^. The collective oscillations of free electrons in metal nanoparticles give rise to electromagnetic resonances (plasmons) that strongly enhance optical signals from nearby molecules^38^,^39^,^40^. To bind plasmonic AuNPs onto the SWNTs, we exploit covalently anchored thiol groups onto the tubes (thiol-functionalized SWNT (SH-SWNT)) as sketched in Fig. 4a, in the Methods section and summarized in Supplementary Fig. 1. TEM microscopy of the resulting Au@SWNT hybrids shows the AuNPs assembled along the sidewall of the SWNTs (see Fig. 4b). This assembly is stable towards environmental changes yet again confirming the covalent nature of the functionalization method.

The functionalization with plasmonic particles markedly increased the luminescence of the SWNTs, which neither happened for defect functionalization nor simple assembly (Supplementary Note 5). The emission of Au@SWNT hybrids in Fig. 4c is two to three times stronger than for uncoated tubes. As only a small fraction of the nanotubes spatially overlaps with the AuNP near field, the increase in the emission cross-section is much higher. Luminescence peak position and width remained constant between pristine and Au@SWNT. The intensity increase originated from an enhanced excitation (absorption) through the strongly localized near-fields around the metallic particle. Interestingly, the maximum enhancement efficiency is obtained for species with excitation windows red-shifted from the AuNP plasmonic resonance (Supplementary Fig. 36), consistent with other coupled emitter-nanometal systems^41^.

The functionalization of carbon nanotubes with dichloro-triazine preserves the electronic and optoelectronic properties of SWNTs. Such functionalized nanotubes are versatile building blocks for nanophotonic hybrids as we demonstrated with two exemplary structures.

## Methods

### Synthesis of the functionalized tubes

HiPCO SWNTs (length: 0.2–1.2 μm, diameter: 0.8–1.2 nm) were purchased from Unidym (batch SP0295). The 2,4,6-trichloro-1,3,5-triazine (cyanuric chloride or triazine), 2,3,3-trimethylindolenine and 5-nitrosalicylaldehyde were purchased from Sigma-Aldrich. Sodium azide and *N*-methyl-2-pyrrolidone were purchased from Merck. A schematic depiction of the reaction steps described below can be found in Supplementary Fig. 1.

### Conjugating triazine onto the SWNTs: SWNT-low and SWNT-high

Pristine SWNTs (1 g) were added to *N*-methyl-2-pyrrolidone (150 ml), sonicated for 1 h and then stirred at room temperature for an additional 1 h. The 2,4,6-1,3,5-trichloro-triazine (10 g, 54 mmol) dissolved in *N*-methyl-2-pyrrolidone (50 ml) was added to the mixture at 0 °C and stirred for 20 min. Sodium azide (1.76 g, 27 mmol) in solid state was gradually added to the reaction flask at 0 °C; the mixture was stirred at this temperature for 2 h. The temperature was raised to 25 °C and stirred for 1 h. Operating at low temperature ensures the substitution of only one chlorine atom^42^,^43^. We thus converted 2,4,6-trichloro-1,3,5-triazine into 2-azido-4,6-dichloro-1,3,5 triazine and prevented the creation of more complicated structures such as di- or tri-azide derivatives, or C_3_N_4_ graphitic materials^44^. Details of the characterization of the intermediate product can be found in the Supplementary Information. Thereafter, the suspension was stirred overnight at a temperature of 25 °C (SWNT-low) and at 70 °C (SWNT-high). The mixtures were centrifuged (5,000 r.p.m. for 5 min) and the crude products were dispersed in acetone and centrifuged again under the same condition. Dispersion and centrifugation of the product was repeated in water, toluene and chloroform to obtain the purified compounds. The products were lyophilized to obtain 1.08 g black solid compound of SWNT-low and 1.03 g black solid compound of SWNT-high.

### Synthesis of SP-SWNTs

The synthesis of the SP derivatives for the SP-SWNTs was performed by the Wagenknecht method^45^. The conjugation of SP onto the SWNTs is a two-step process requiring first the attachment of the indole group onto the triazine to be used for the growth of the SP. SWNT-low (0.2 g) was dispersed in *N*-methyl-2-pyrrolidone (150 ml) and sonicated for 1 h. The 2,3,3-Trimethylindolenine (2 ml, 12.47 mmol) dissolved in *N*-methyl-2-pyrrolidone (10 ml) was added to this mixture at 0 °C and stirred for 1 h. The mixture was sonicated at room temperature for 1 h and the temperature was raised to 65 °C. Finally, the mixture was stirred under nitrogen atmosphere for 4 days. It was centrifuged at 7,000 r.p.m. for 10 min, washed by acetone, chloroform, tetrahydrofuran (THF) and water, and collected by centrifugation. The product was lyophilized overnight obtaining 0.19 g black solid compound of SWNT-indole. The intermediate step required SWNT-indole to be added to a saturated solution of NaOH in water and sonicated for 30 min. The mixture was stirred for 5 h at room temperature and the final product (SWNT-indolene) was purified by repeated washing with water and centrifugation. For the characterization of the reaction of dichlorotriazene with dimethyl indoline, please refer to Supplementary Note 1. The final step required to add an excess amount of 5-nitrosalicylaldehyde (2.5 g, 1.19 mmol) at 25 °C to a well-sonicated and degassed dispersion of SWNT-indolene (0.1 g) in dry ethanol (70 ml). The mixture was sonicated at 25 °C with 35 kHz for 2 h and stirred at 70 °C overnight. Finally, the solvent was evaporated, the mixture dispersed in ethanol, chloroform, water, toluene and acetone, and collected by centrifugation at 5,000 r.p.m. for 5 min.

### Synthesis of SH-SWNT

Based on the protocols for the nucleophilic substitution of the chlorine atoms of the triazine reported in literature, cysteine was conjugated to the triazine functional groups^42^,^43^,^46^. SWNT-high (100 mg) was dispersed in dimethylfomamide (50 ml) and sonicated for 15 min at room temperature. Next, cysteine (1 g, 8.2 mmol) and triethylamine (1.72 ml, 12.3 mmol) were added to the mixture that was stirred at 65 °C for 2 days. The mixture was then dialysed for 1 week in water. We lyophilized the product and 90 mg black compound of SH-SWNT was obtained.

### Sample preparation

To solubilize the carbon nanotubes, we dissolved them in water (density of tubes 0.1 g l^−1^) and added sodium cholate (SC, 1 wt %). Following the routine described in our past works^47^,^48^, the solution was sonicated with a tip-sonicator (Bandelin Sonopuls HD 2070) for 1 h at 60 W and then centrifuged (Hettich Mikro 220 R centrifuge) at 30,000 *g* for 1 h. The supernatant was collected and used for optical measurements. For the functionalized nanotubes, we corrected the values of the density of functionalized nanotubes dissolved in water by taking into account the mass change due to the attachment of the functionalities (fraction of SWNT enlisted in Supplementary Table 4).

The AuNPs were synthesized according to the Turkevich method^49^. We obtained spherical AuNPs with diameters 10–20 nm. The plasmon absorption band peaked at 520 nm (Supplementary Fig. 34). The Au@SWNT hybrids were created by adding 200 μl of 0.1 g l^−1^ thiol-SWNTs into 1,000 μl of the AuNP solution. The mixture was stirred overnight. To promote further debundling we added SC (1 wt%) to the solutions and applied mild sonication for 1 h at ≈20 W. After decantation, the supernatant was used for spectroscopic characterization. For TEM imaging of the Au@SWNTs, we dropped 5 μl of the solution onto a nickel grid covered with a lossy carbon film. The grid was placed over a heating plate until complete evaporation of the liquid.

### Experimental details

Two-dimensional luminescence was recorded with a Nanolog spectrofluorometer from Horiba, equipped with a Xenon lamp and a liquid-Nitrogen cooled InGaAs detector. Ultraviolet–visible–infrared absorption spectra were taken with a Perkin-Elmer Lambda 950 spectrophotometer. Kinetic absorption measurements under ultraviolet illumination were performed with a spectrophotometer from Thermo-Fisher. The ultraviolet light source was a handheld ultraviolet lamp emitting at 365 nm. The Raman spectra were acquired with a Horiba LabRam spectrometer equipped with an He-Ne laser (633 nm). The TEM measurement of the Au@SWNTs were taken with a transmissions electron microscope FEI Tecnai G^2^ 20 S-TWIN with LaB6-cathode, 120 kV acceleration voltage and a GATAN MS794 CCD acquistion camera, with 1,024 × 1,024 pixels and point resolution of 0.24 nm. Details on the X-ray photoelectron spectroscopy as well as on the HR(S)TEM and EELS setups can be found in the Supplementary Methods.

### Quantum chemical calculations

Calculations were performed using MOPAC2016 ( http://openmopac.net/). The geometries of the (8,0) nanotube, the nitrenes and the nanotube-addend (nitrenes) systems were fully optimized at the PM7 level using a minimal basis set^50^. The climbing image nudged elastic band method^51^ was used to determine transition state configurations and barrier energies for the cycloaddition reaction (see Supplementary Note 2 for details).

### Data availability

The data supporting the findings of this study are available within the article and its Supplementary Information files, and from the corresponding author upon reasonable request.

## Additional information

**How to cite this article:** Setaro, A. *et al*. Preserving *π*-conjugation in covalently functionalized carbon nanotubes for optoelectronic applications. *Nat. Commun.* **8,** 14281 doi: 10.1038/ncomms14281 (2017).

**Publisher's note:** Springer Nature remains neutral with regard to jurisdictional claims in published maps and institutional affiliations.

## Supplementary Material

Supplementary Information Supplementary Figures, Supplementary Tables, Supplementary Notes, Supplementary Methods and Supplementary References.
