# Supplementary material for: Preserving π-conjugation in covalently functionalized carbon nanotubes for optoelectronic applications
Source: Nat Commun. 2017 Jan 30;8:14281. doi: 10.1038/ncomms14281 (PMC5290266; doi:10.1038/ncomms14281)
Supplement: Supplementary Information — Supplementary Figures, Supplementary Tables, Supplementary Notes, Supplementary Methods and Supplementary References. [file ncomms14281-s1.pdf]

Supplementary Figures

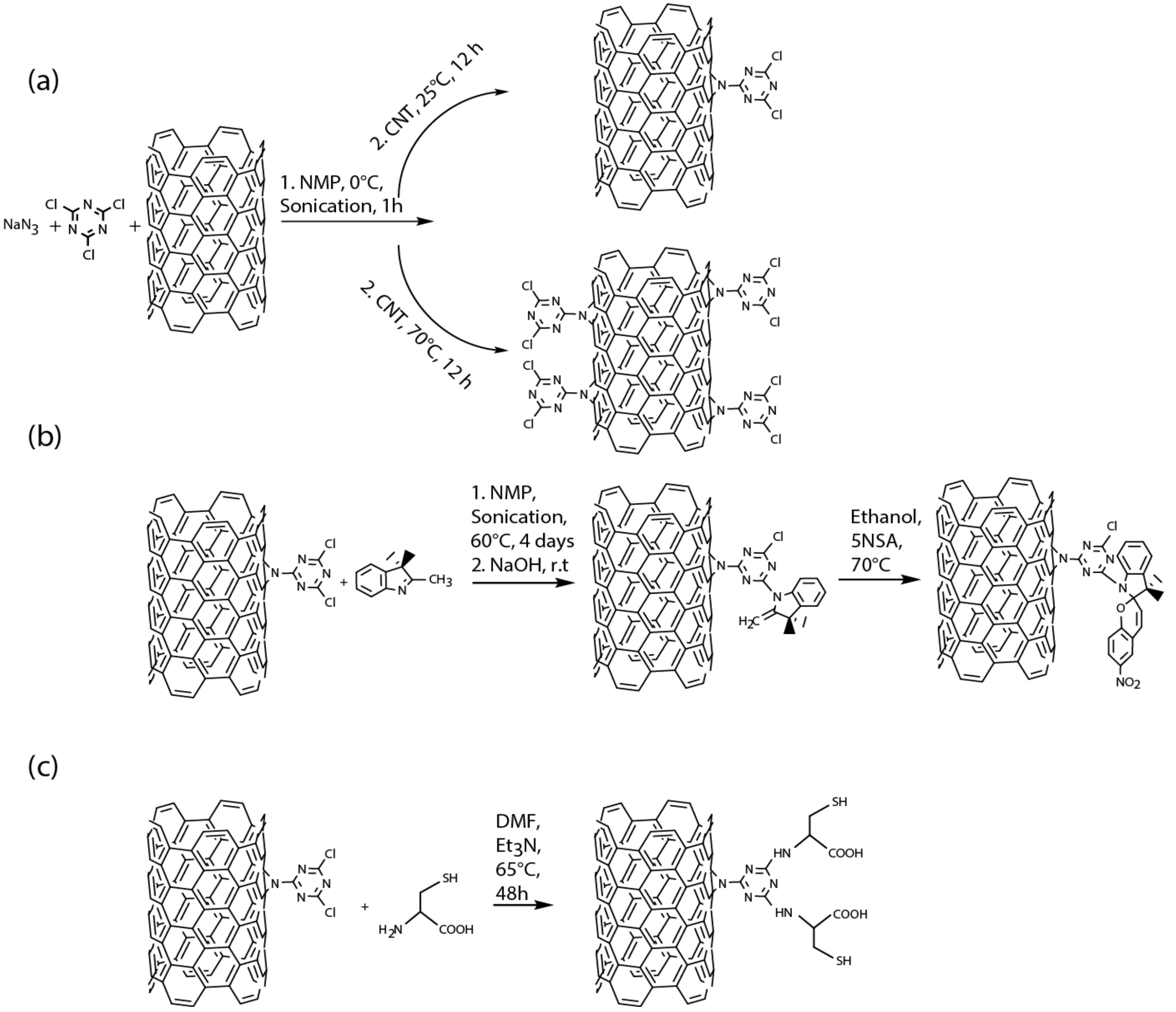

Supplementary Figure 1. Molecular sketches of the synthesis of: (a) SWNT-low and SWNT-high, (b) SP-SWNT via SWNT-indole, and (c) SH-SWNT.

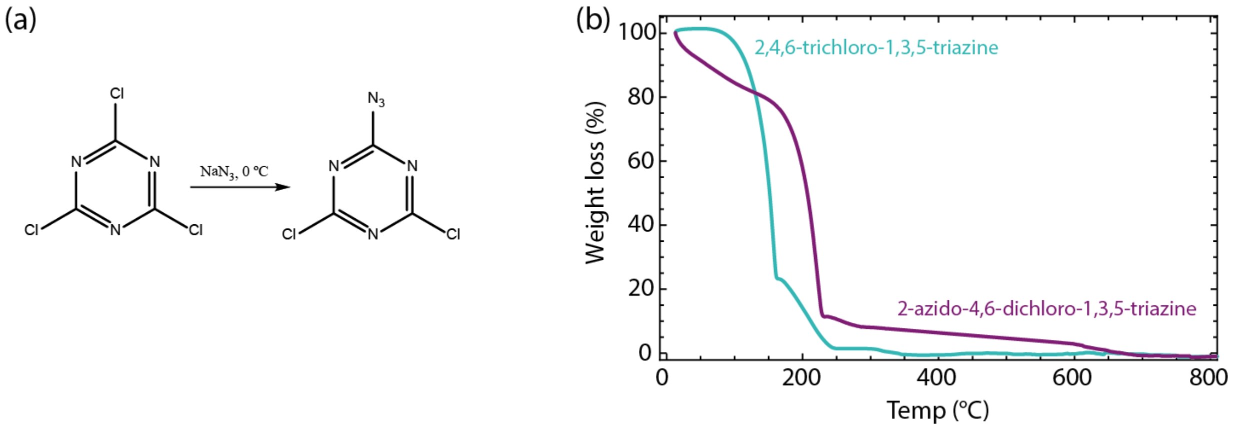

Supplementary Figure 2. (a) The reaction between sodium azide and 2,4,6-trichloro-1,3,5-triazine at low temperature results in the formation of 2-azido-4,6-dichloro-1,3,5-triazine. (b) TGA thermograms of 2,4,6-trichloro-1,3,5-triazine and 2-azido-4,6-dichloro-1,3,5-triazine.

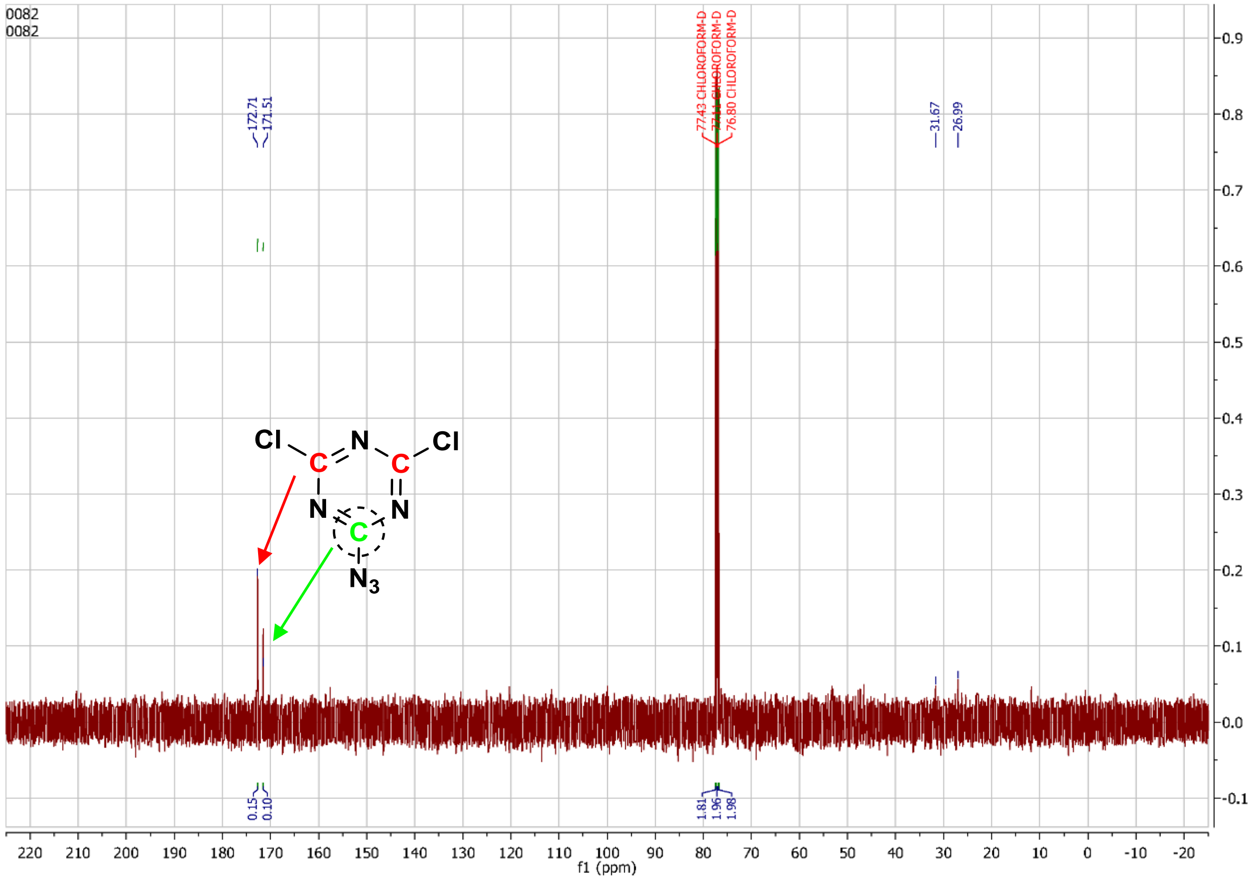

Supplementary Figure 3. ^{13}C NMR spectra of 2-azido-4,6-dichloro-1,3,5-triazine in $CDCl_3$.

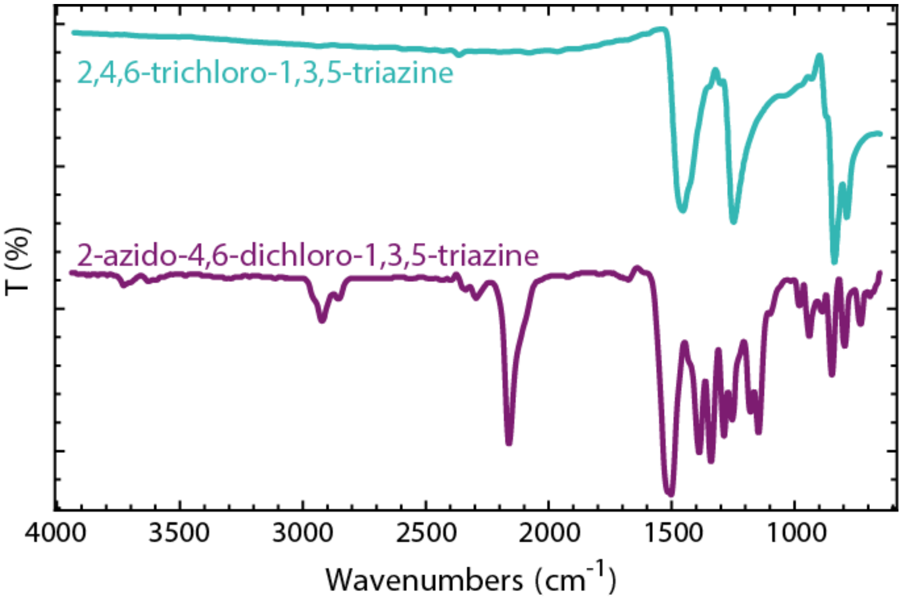

Supplementary Figure 4. IR spectra of 2,4,6-trichloro-1,3,5-triazine and 2-azido-4,6-dichloro-1,3,5-triazine.

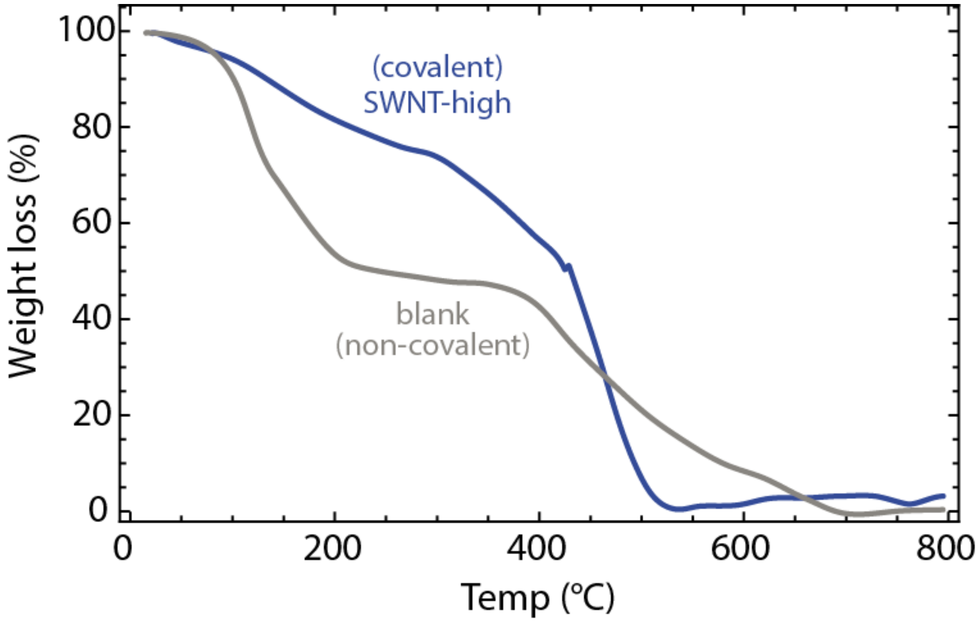

Supplementary Figure 5. TGA thermogram of mixture of SWNTs and 2,4,6-1,3,5-trichloro-triazine (blank) and SWNT-high (SWNT-high).

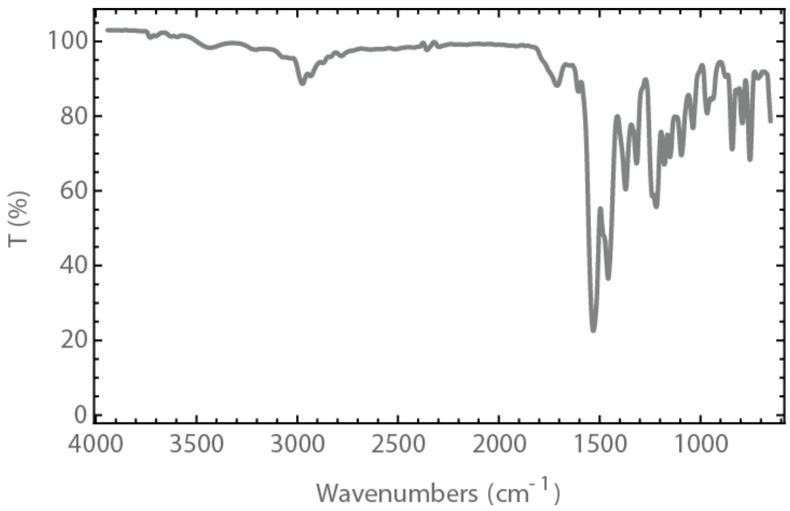

Supplementary Figure 6. IR spectra of 1-(4,6-dichloro-1,3,5-triazine)-2,3,3-trimethylindolinium chloride.

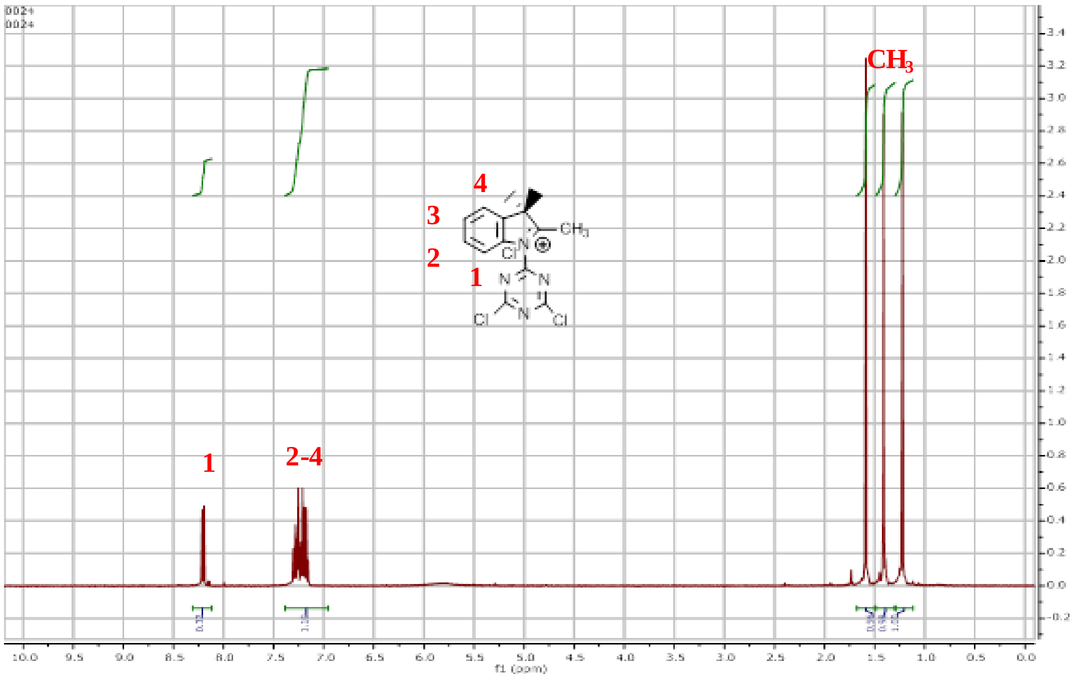

Supplementary Figure 7. ^1H NMR spectra of 1-(4,6-dichloro-1,3,5-triazine)-2,3,3-trimethylindolinium chloride in $CDCl_3$.

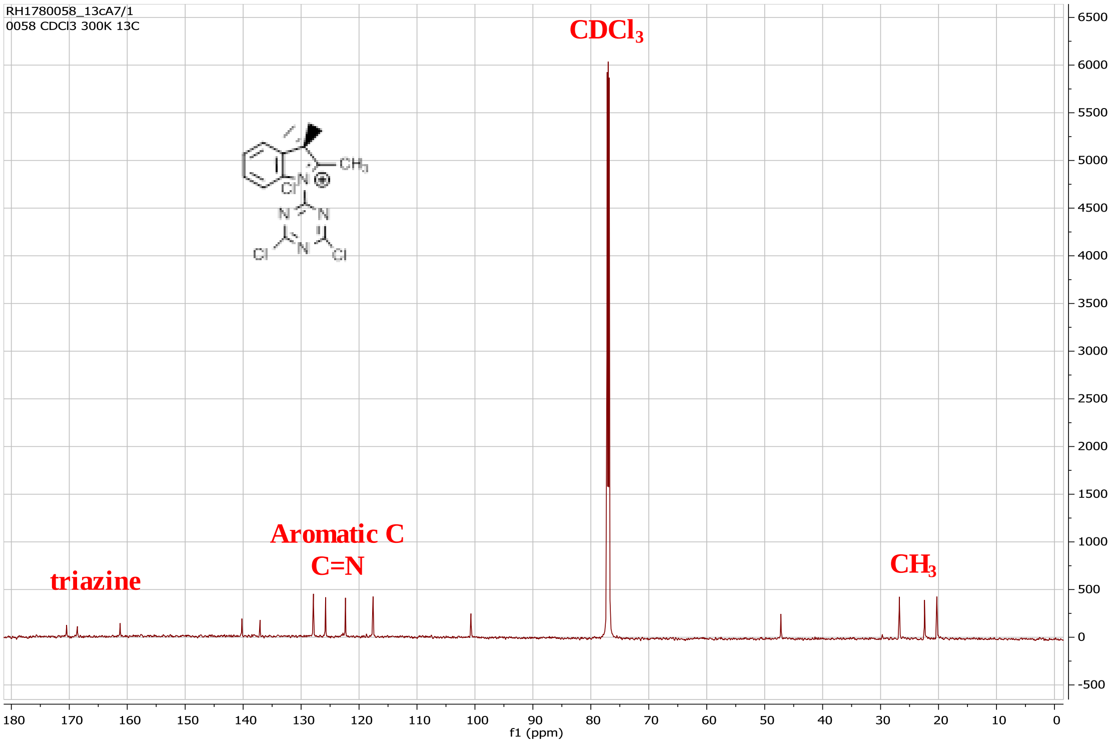

Supplementary Figure 8. ^{13}C NMR spectra of 1-(4,6-dichloro-1,3,5-triazine)-2,3,3-trimethylindolinium chloride in $CDCl_3$.

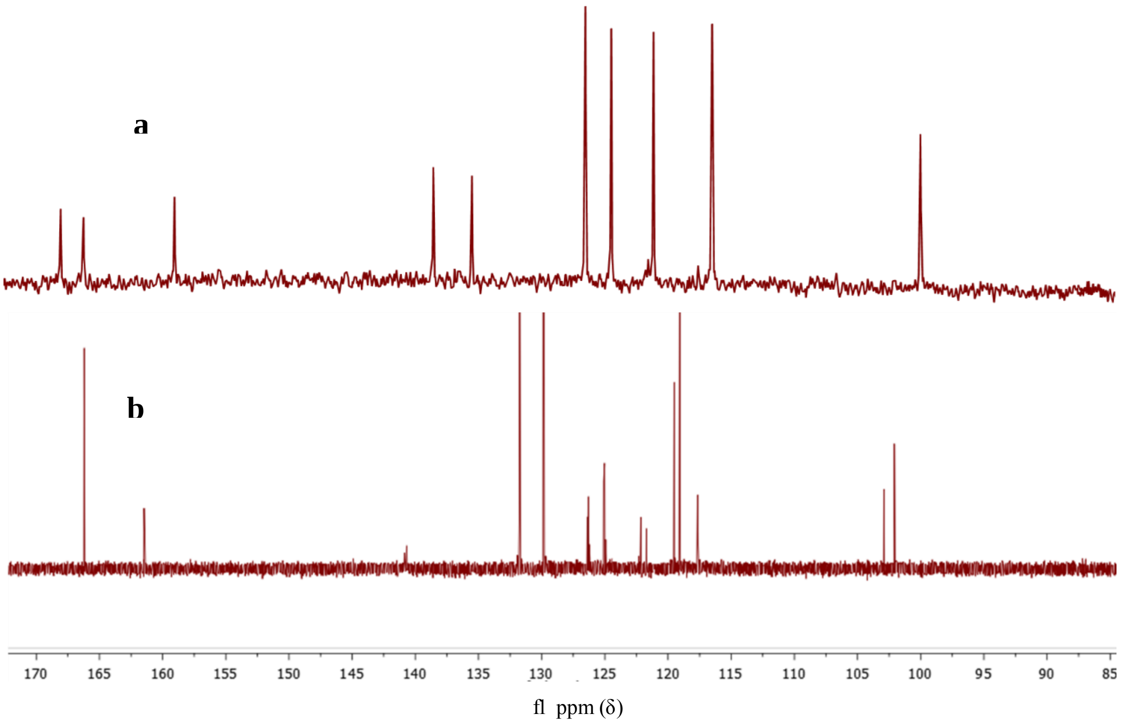

Supplementary Figure 9. ^{13}C NMR spectra of (a) 1-(4,6-dichloro-1,3,5-triazine)-2,3,3-trimethylindolinium chloride and (b) Synthesis of (R)-6-(3',3'-dimethyl-6-nitrospiro[chromene-2,2'-indolin]-1'-yl)-1,3,5-triazine-2,4-diol.

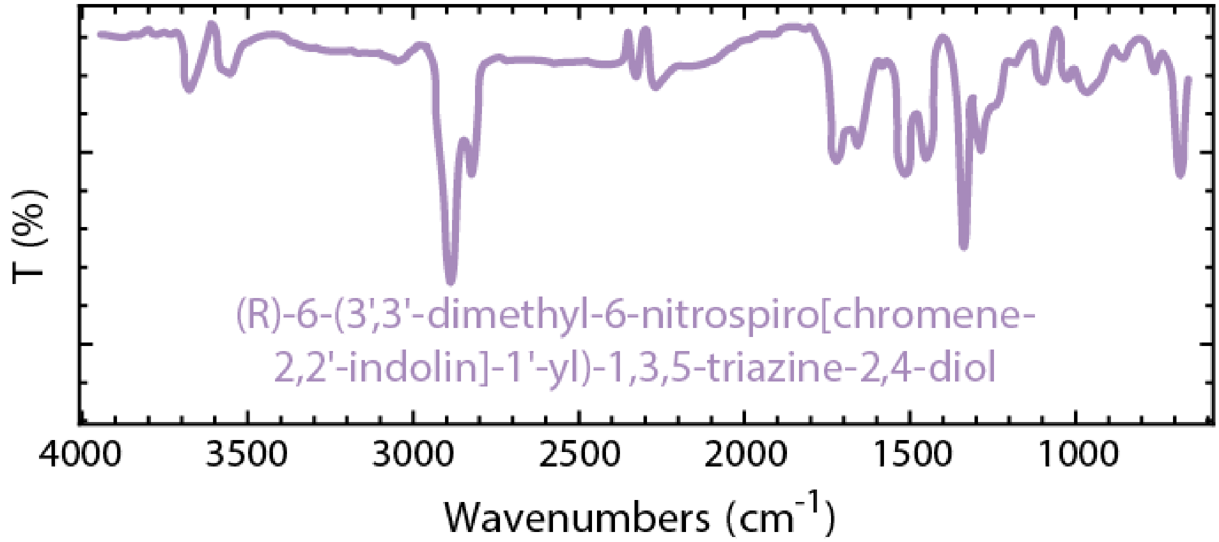

Supplementary Figure 10. IR spectrum of (R)-6-(3',3'-dimethyl-6-nitrospiro[chromene-2,2'-indolin]-1'-yl)-1,3,5-triazine-2,4-diol.

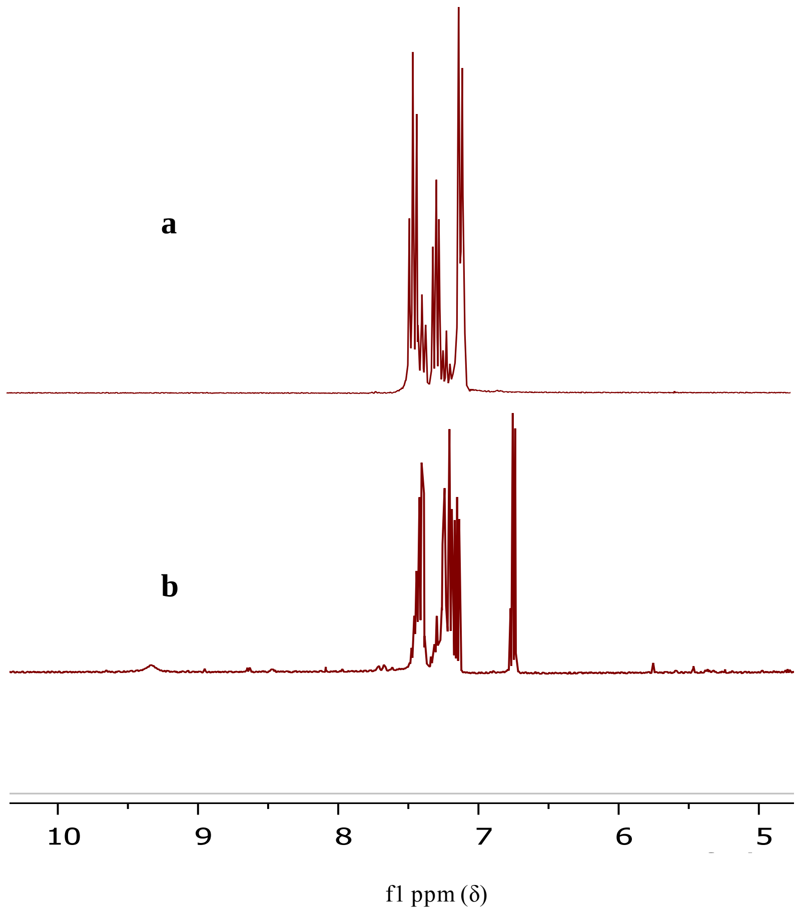

Supplementary Figure 11. ^{1}H NMR spectra of (a) 2-chloro-4,6-diphenoxy-1,3,5-triazine and (b) (4,6-diphenoxy-1,3,5-triazin-2-yl)-L-cysteine.

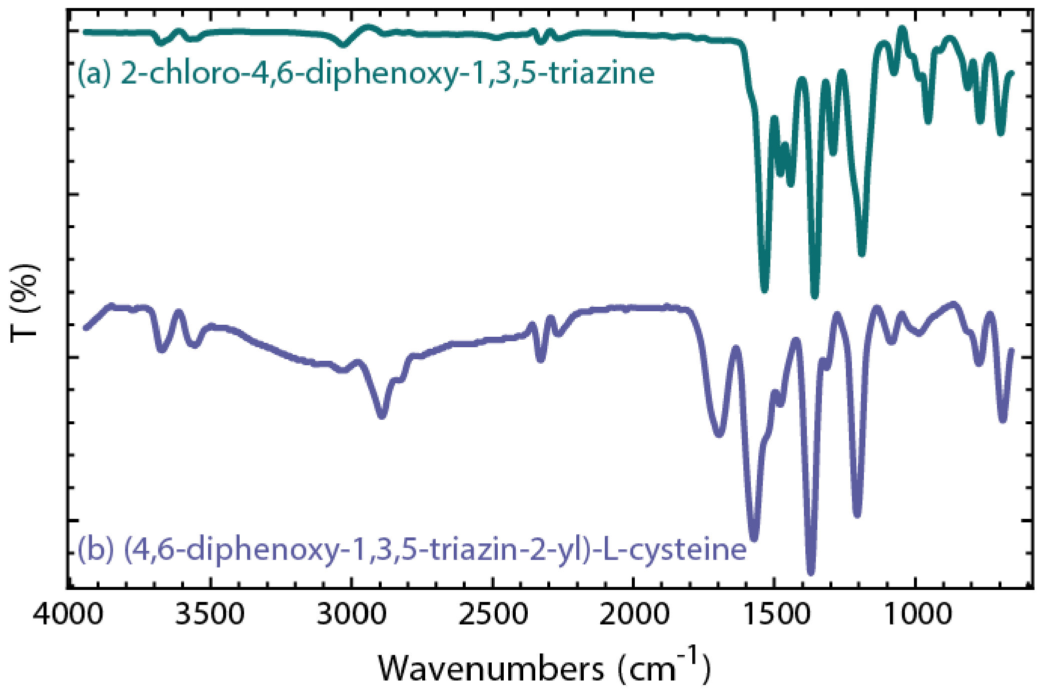

Supplementary Figure 12. IR spectra of a) 2-chloro-4,6-diphenoxy-1,3,5-triazine and b) (4,6-diphenoxy-1,3,5-triazin-2-yl)-L-cysteine.

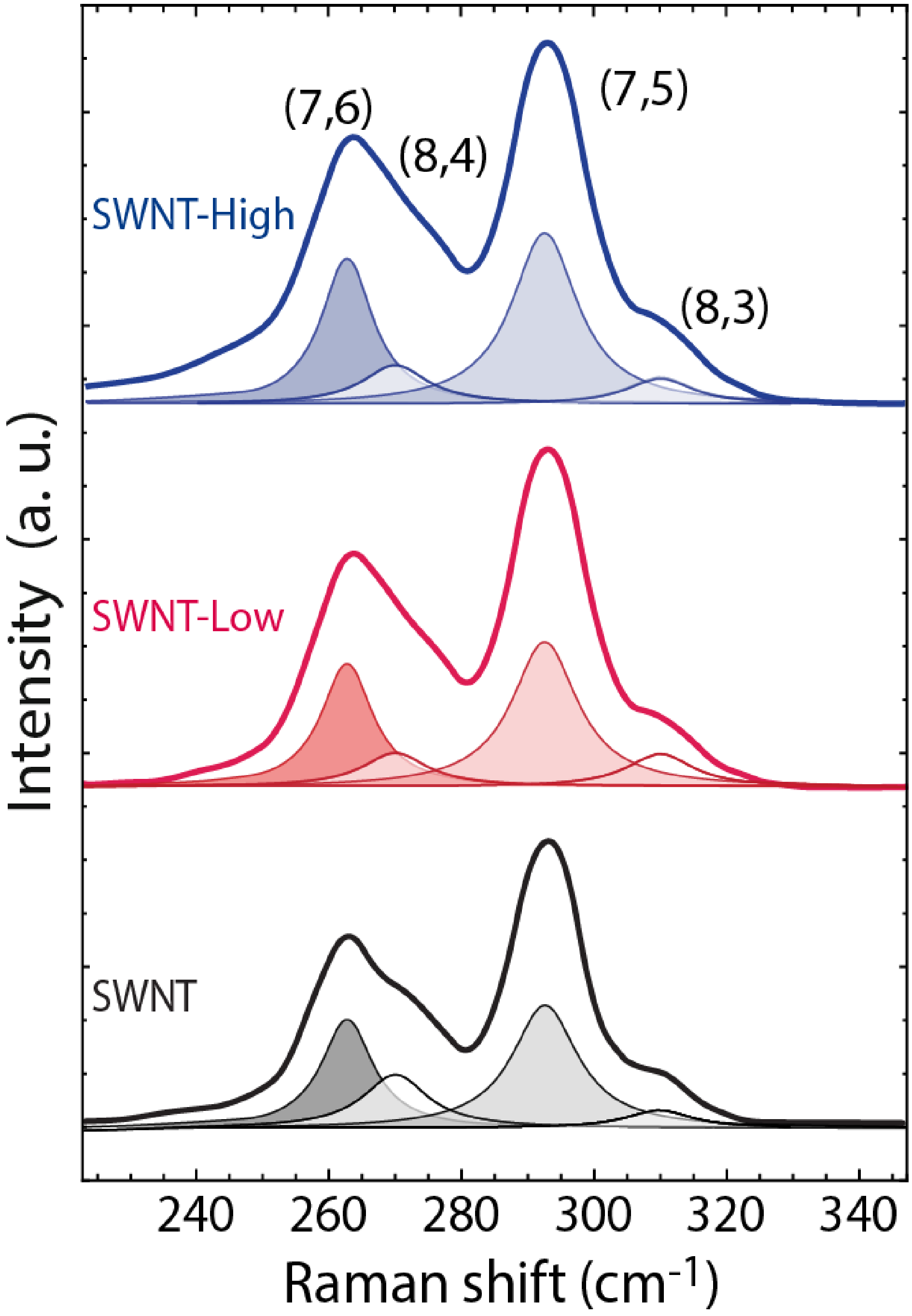

Supplementary Figure 13. Raman spectra in the RBM region of pristine and triazine-functionalized nanotubes.

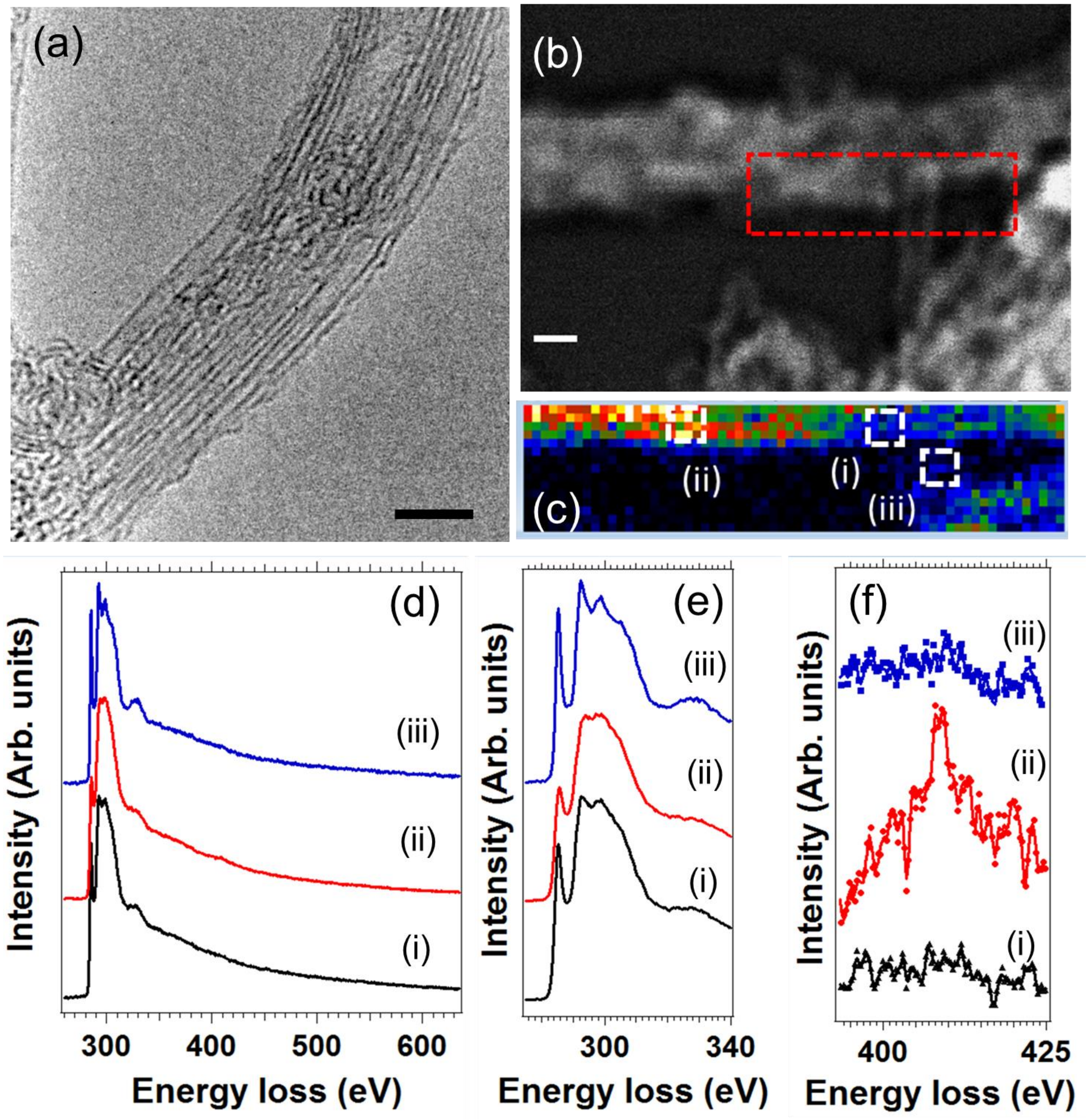

Supplementary Figure 14. (a) HRTEM micrograph of a bundle of single-walled nanotubes (SWNT-high sample). The scale bar is 5 nm. (b) HAADF-STEM image of another rope of 3 or 4 single-walled CNTs. An EELS SPIM has been recorded in the red marked area of this image. The scale bar is 2 nm. (c) Nitrogen map extracted from the EELS-SPIM. (d) Sums of 9 selected EEL spectra collected from the three different marked areas in the SPIM, Fig. 14b. While the C-K edge is visible in all the spectra, the N-K edge is only visible in the red (Fig. 14d-(ii)) EEL spectrum. (e) and (f) C- and N-K edges extracted from the EEL spectra of the Fig. 14d, respectively. To filter noise, spectra of Fig. 14f were smoothed using a Savitzky−Golay filter (second-order polynomial).

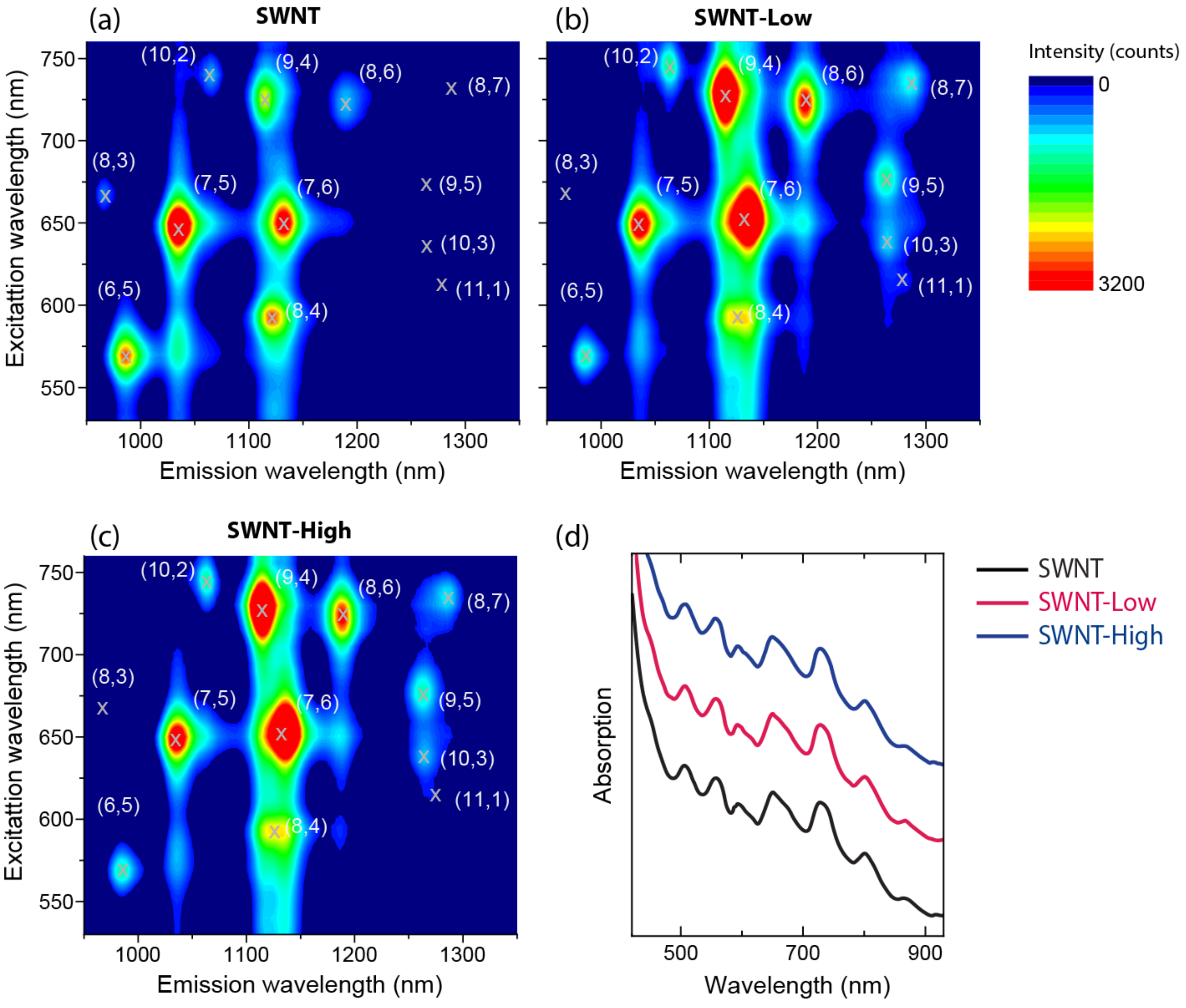

Supplementary Figure 15. Excitation-emission maps of the (a) pristine SWNTs, (b) SWNT-low and (c) SWNT-high. (d) Absorption spectra of the SWNT samples.

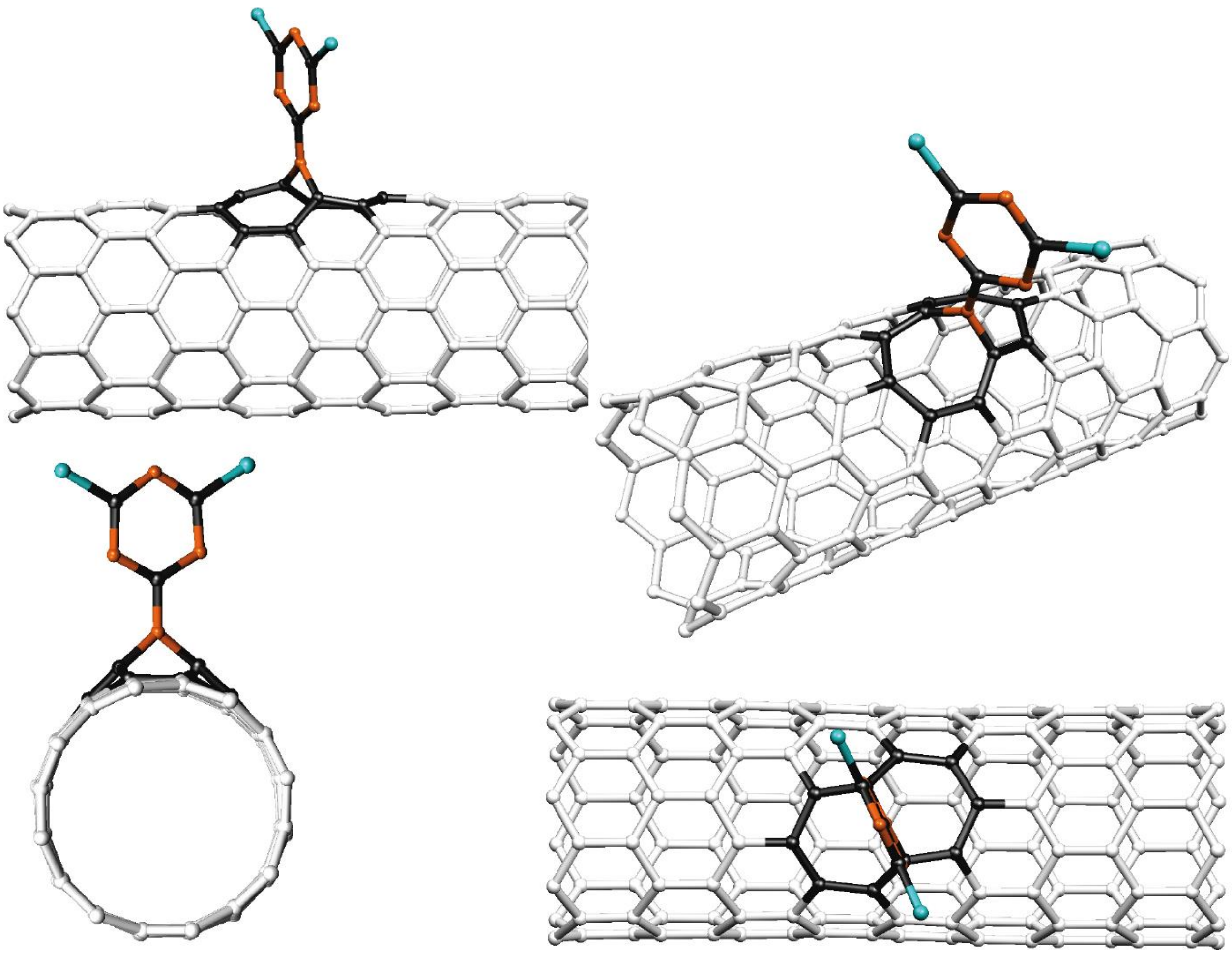

Supplementary Figure 16. Molecular configuration of the triazine on an (8,0) nanotube under different points of view and perspectives. Carbon atoms of the triazine and CNT atoms close to the triazine are highlighted in black. Hydrogen atoms were omitted for clarity.

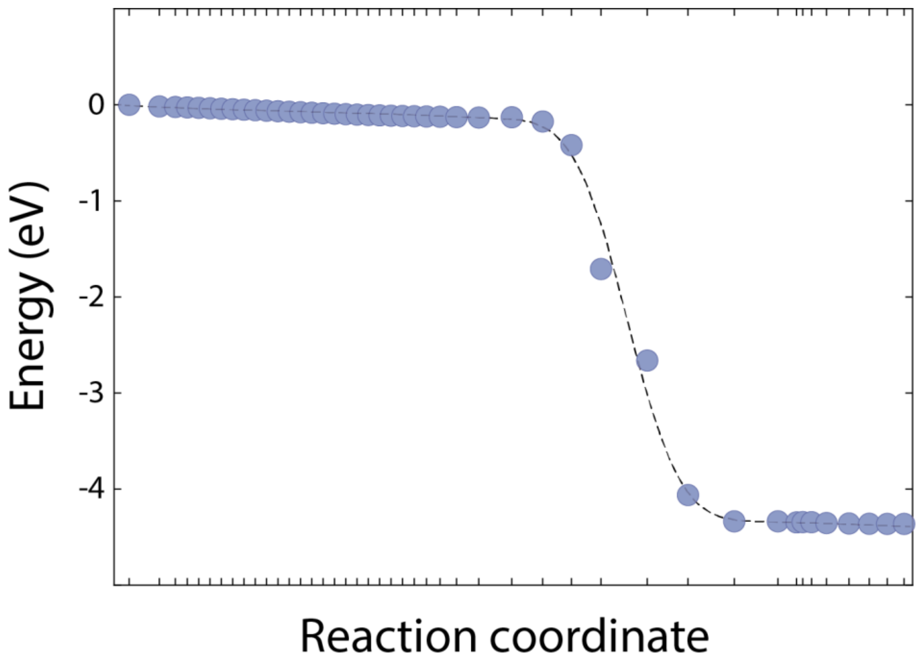

Supplementary Figure 17. Energy profile for the MEP of the cycloaddition with the CI-NEB method.

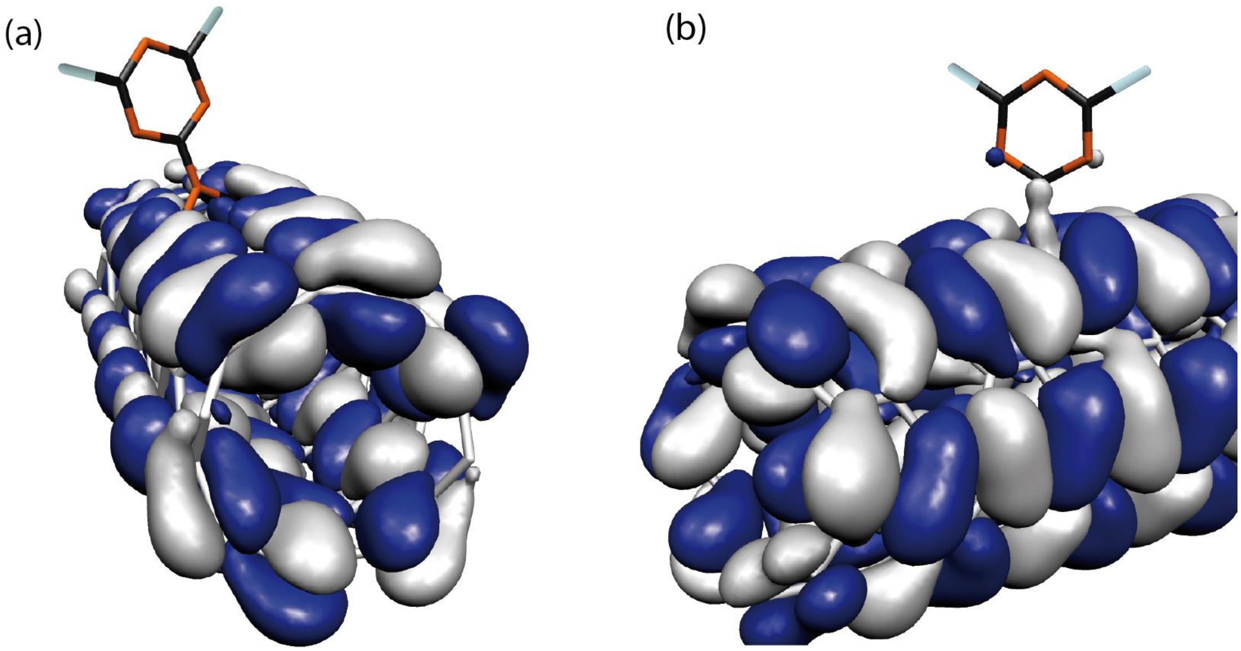

Supplementary Figure 18. HOMO of the triazine-functionalized (8,0) nanotube for the isosurface at (a) $7.6\cdot10^{-3}e^{-}$ nm^{-3} and (b) $2.5\cdot10^{-3}e^{-}$ nm^{-3}.

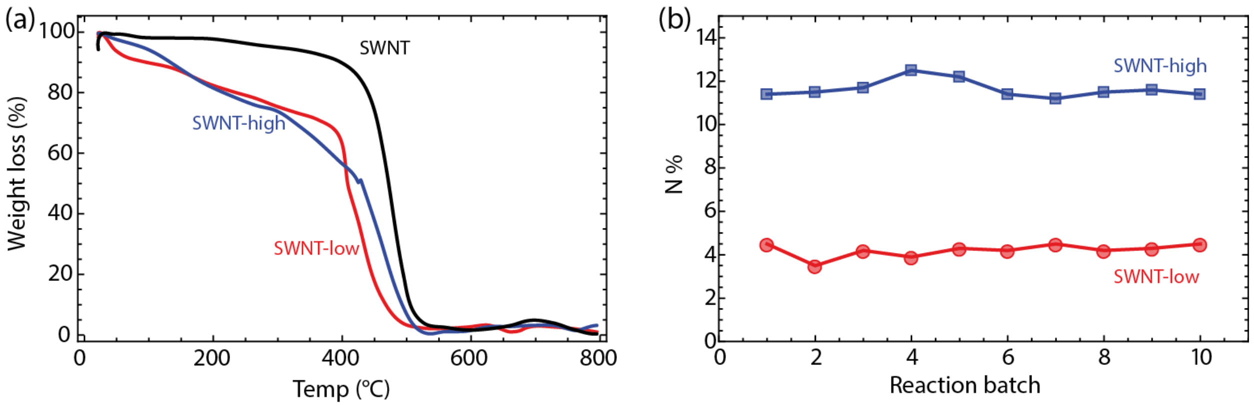

Supplementary Figure 19. (a) TGA diagrams for pristine nanotubes (black curve), SWNT-low (red curve) and SWNT-high (blue curve), at air atmosphere. (b) Variation in the nitrogen content of SWNT-low and SWNT-high obtained from ten different reaction batches.

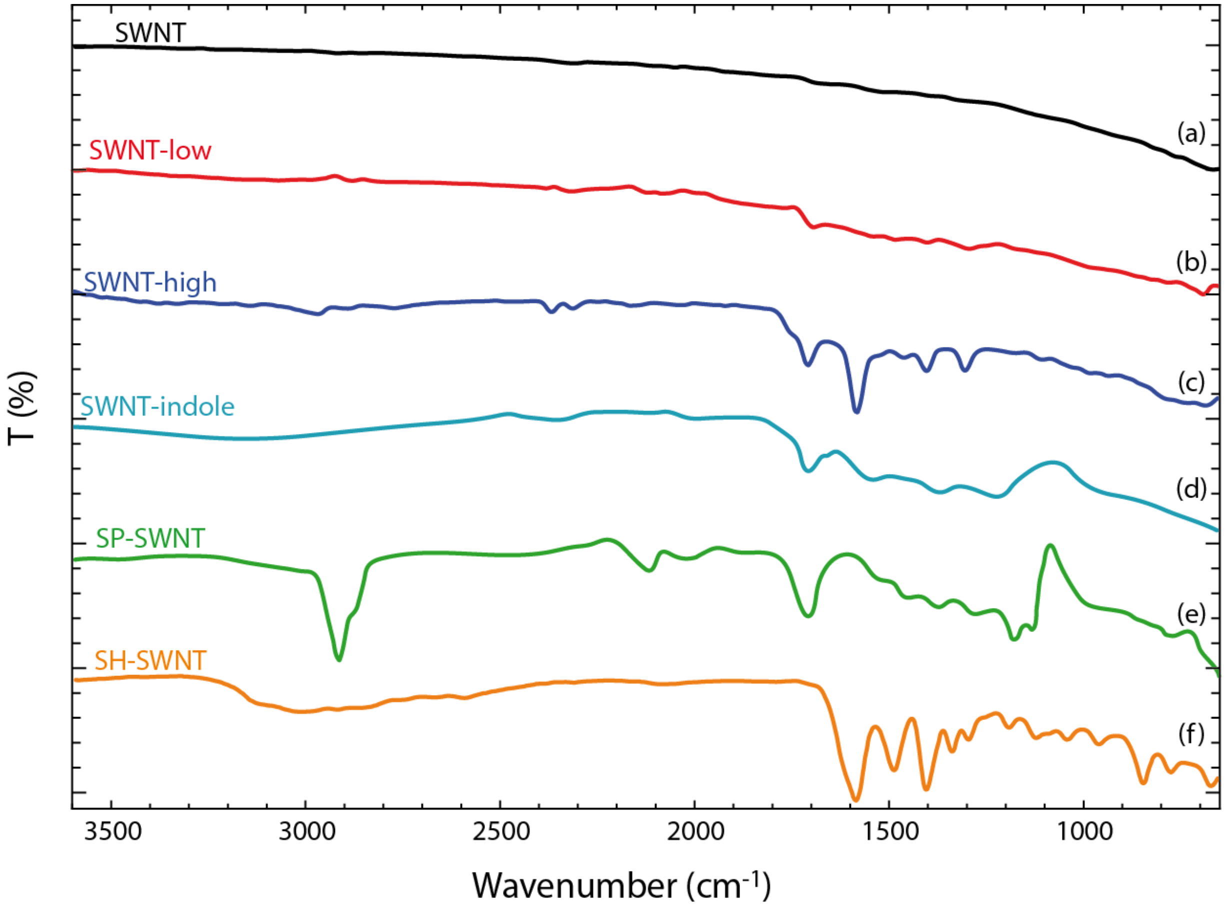

Supplementary Figure 20. IR spectra of the functionalized SWNT samples (see Supplementary Figure 1): (a) pristine SWNT, (b) SWNT-low, (c) SWNT-high, (d) SWNT-indole, (e) SP-SWNT, and (f) SH-SWNT.

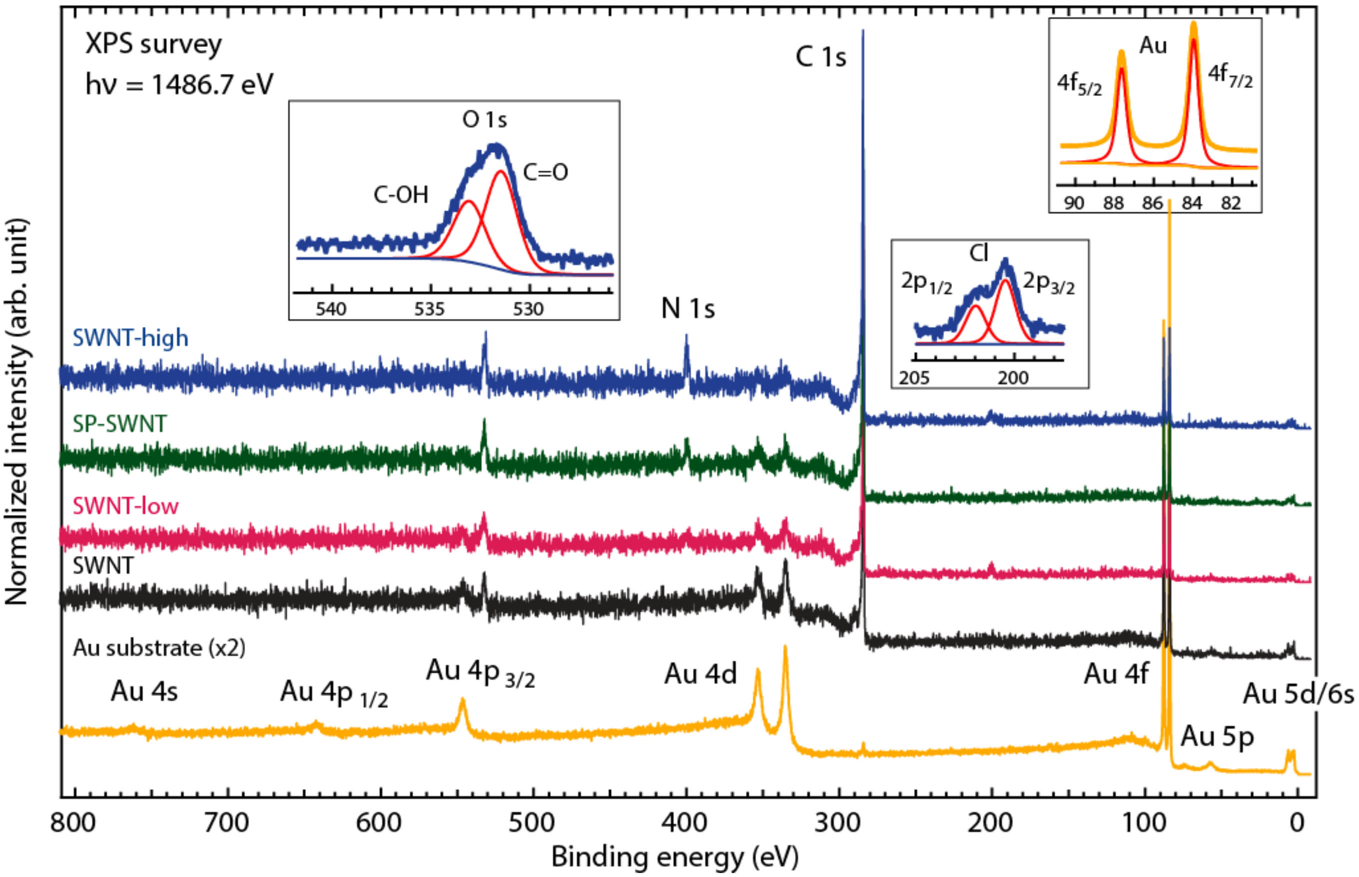

Supplementary Figure 21. Survey spectra of the functionalized SWNTs along with pristine SWNT and a reference Au substrate. All SWNT spectra are normalized on the sp^2-hybridized C 1s component of SWNT. The Au spectrum is normalized to twice the Au 4f peaks of SWNT. Insets show selected spectral details.

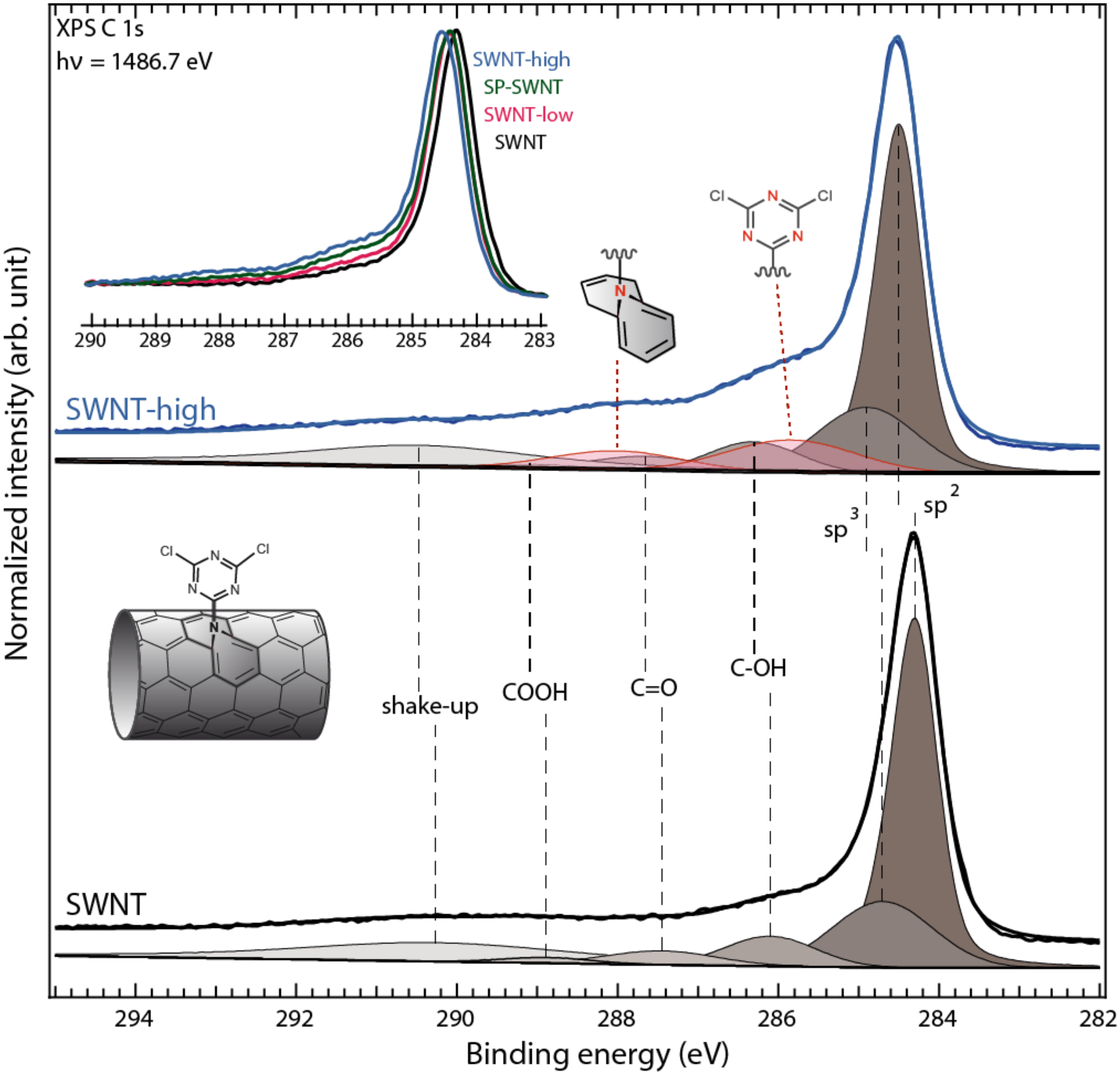

Supplementary Figure 22. C 1s XP spectra of SWNT and SWNT-high along with their spectral decomposition. All spectra are normalized on their respective sp^2 components. The inset shows that the closely related C 1s XP spectra of the SWNT samples shift to higher binding energy with increasing covalent functionalization.

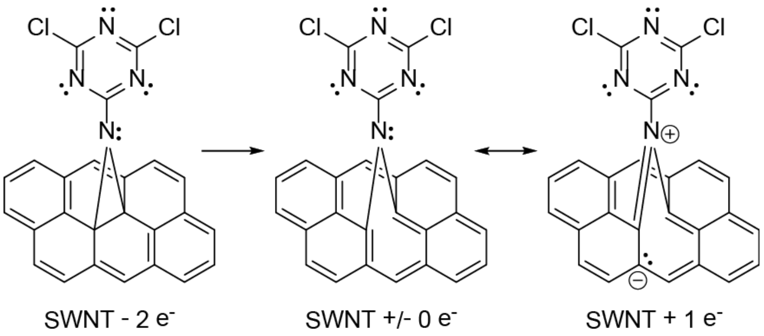

Supplementary Figure 23. Restoration of the sp^2-conjugated π-electron system of the SWNT by ring opening of the aziridine moiety after covalent functionalization. After that the electron lone pair of the bridging nitrogen atom connects the conjugated π-electron systems of the SWNT and the triazine unit. By mesomerism the electron density in the SWNT is increased.

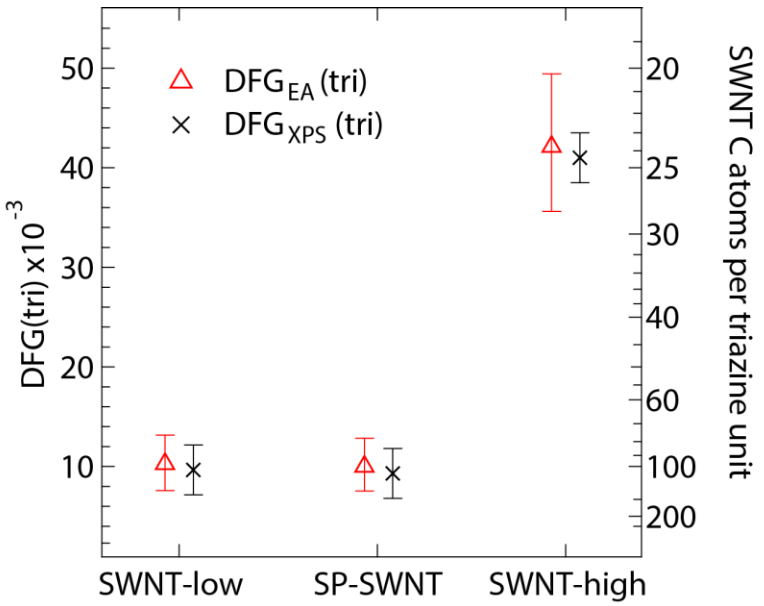

Supplementary Figure 24. Determination of the degree of functionalization: Comparison between elemental analysis and XPS results.

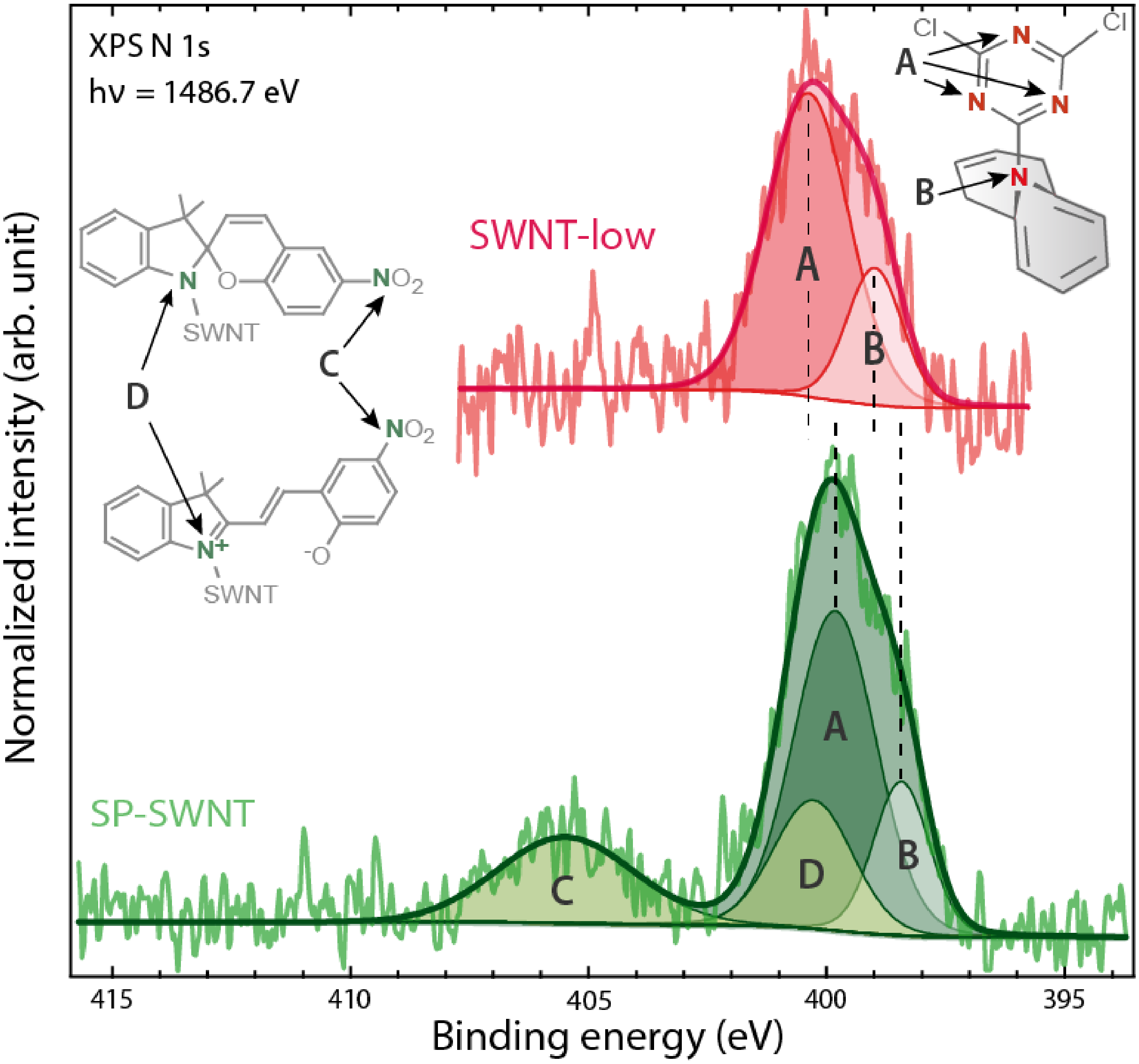

Supplementary Figure 25. N 1s XP spectra of SWNT-low and SP-SWNT along with their spectral decompositions. The triazine peaks are equal in both spectra, but shifted with respect to each other. The combined peak area for the indoline/indole iminium cation is set equal to that of NO_2.

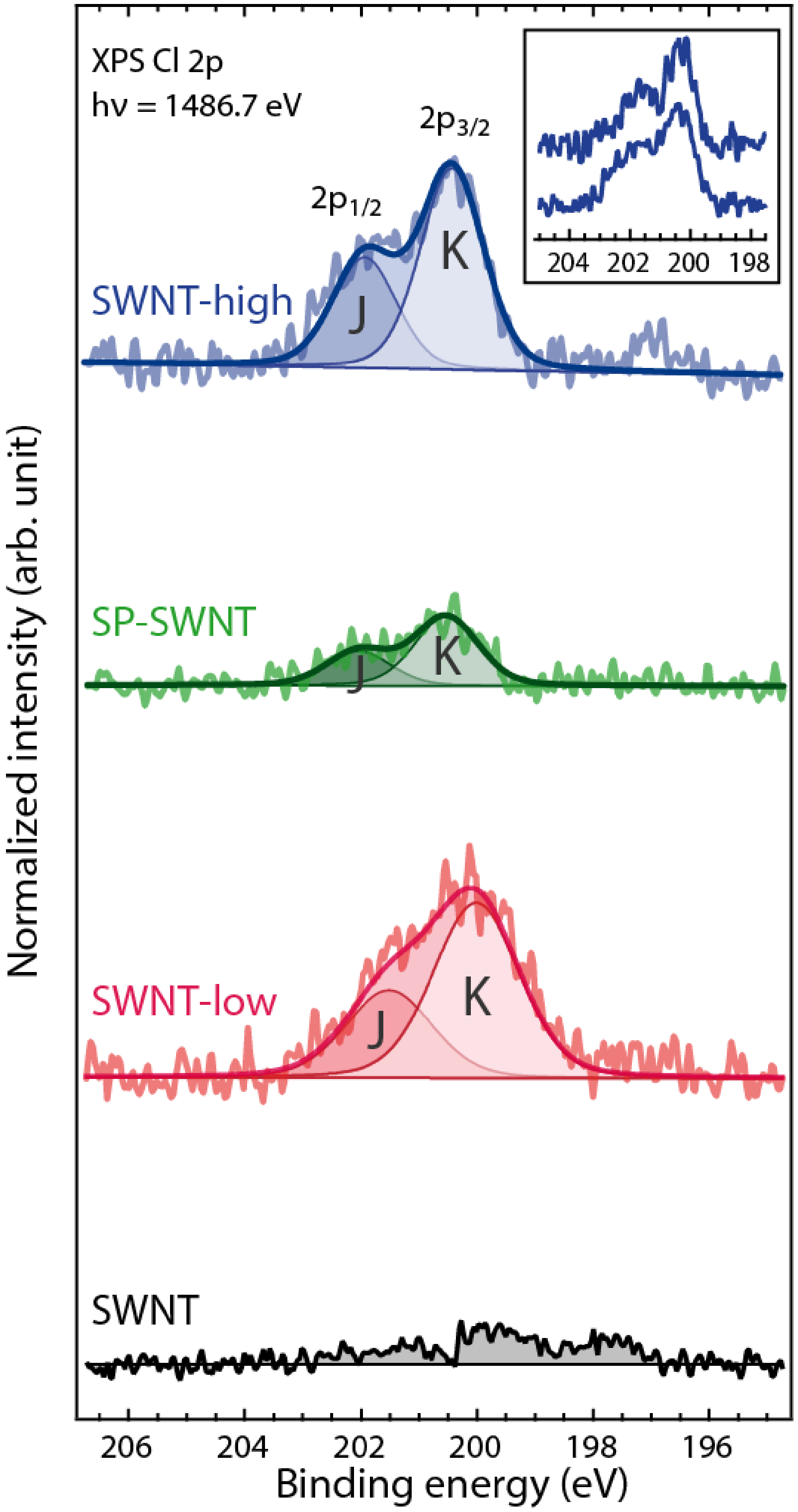

Supplementary Figure 26. Cl 2p XP spectra of pristine and functionalized SWNTs. The inset shows two subsequent measurements on the same sample: No observable X-ray beam-induced Cl loss occurs. The small artifact in SWNT-high around E_B = 197 eV is ascribed to additional residual Cl contaminations from the solvent.

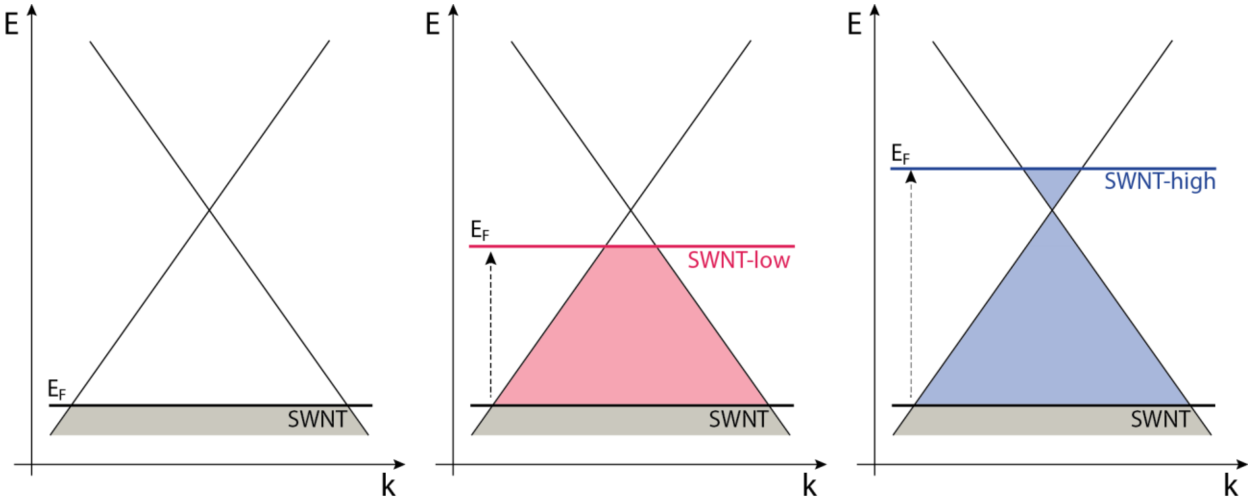

Supplementary Figure 27. Schematic illustration of the functionalization effect onto the Fermi energy of the nanotubes.

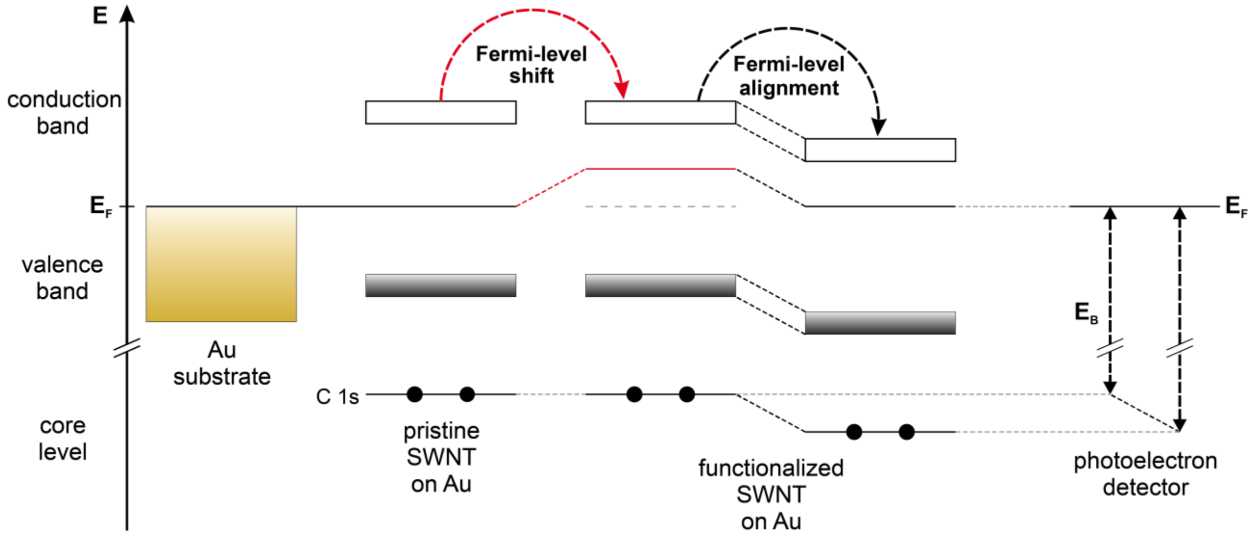

Supplementary Figure 28. XPS schematic of the C 1s peak shift upon SWNT functionalization.

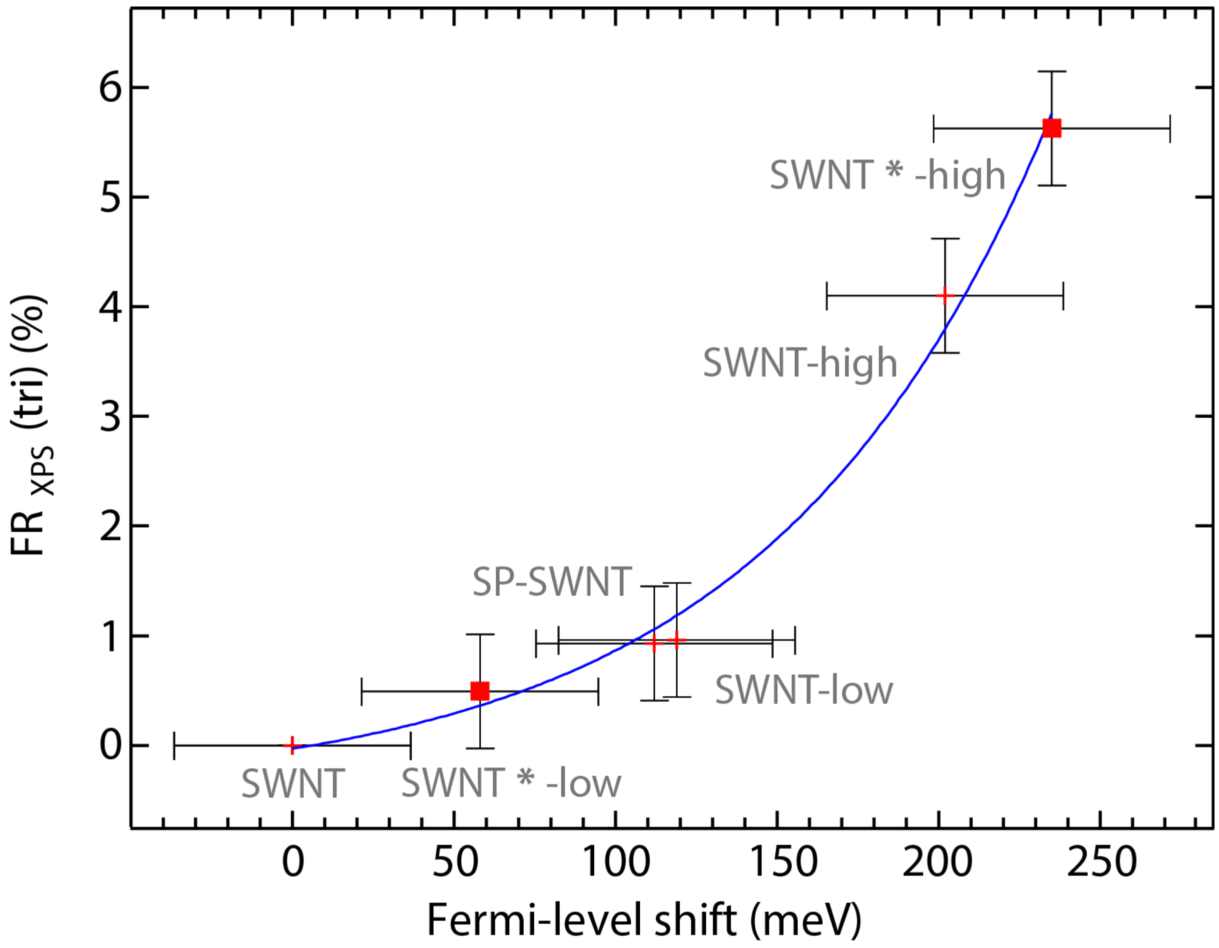

Supplementary Figure 29. Plot of the functionalization ratios of the triazine-unit FR_{XPS}(tri) against Fermi-level shifts obtained by comparing the spectral shifts of the XPS C 1s peaks of functionalized SWNTs with the pristine SWNTs (Supplementary Table 5). The data were fitted with the exponential model $FR_{XPS} \sim \exp[\beta^{-1}\,\Delta E]$ with β=(90±10)meV.

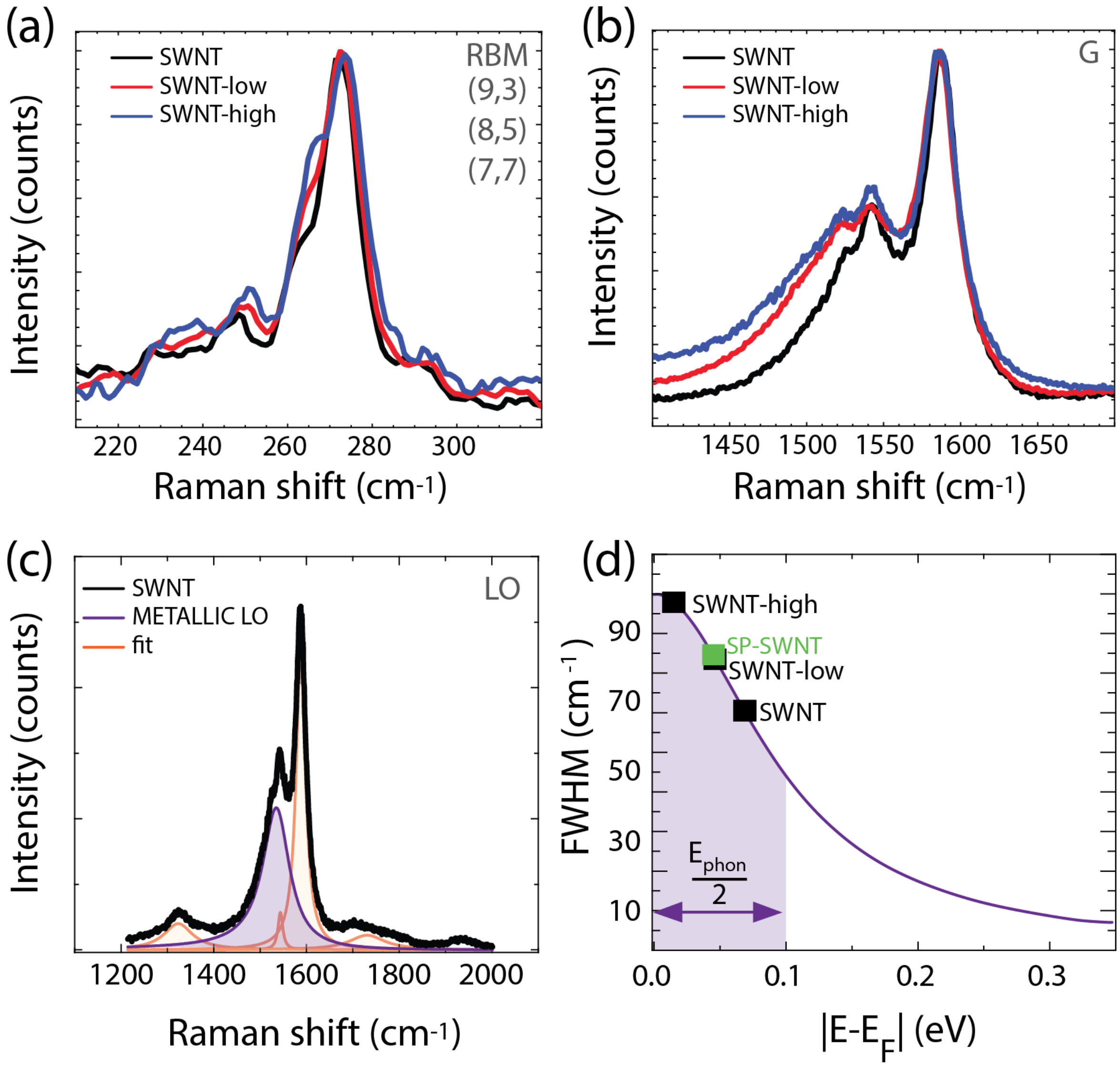

Supplementary Figure 30. RBM (a) and G (b) spectra of the three samples with different functionalization levels. The LO position shifts to lower energies by 14 cm^{-1}, the linewidth increases by 30 cm^{-1}. (c) G band fit of the SWNT sample, the metallic LO band is highlighted in purple. (d) Metallic LO broadening by shift of the Fermi level, FWHM is adjusted to the phonon energy.

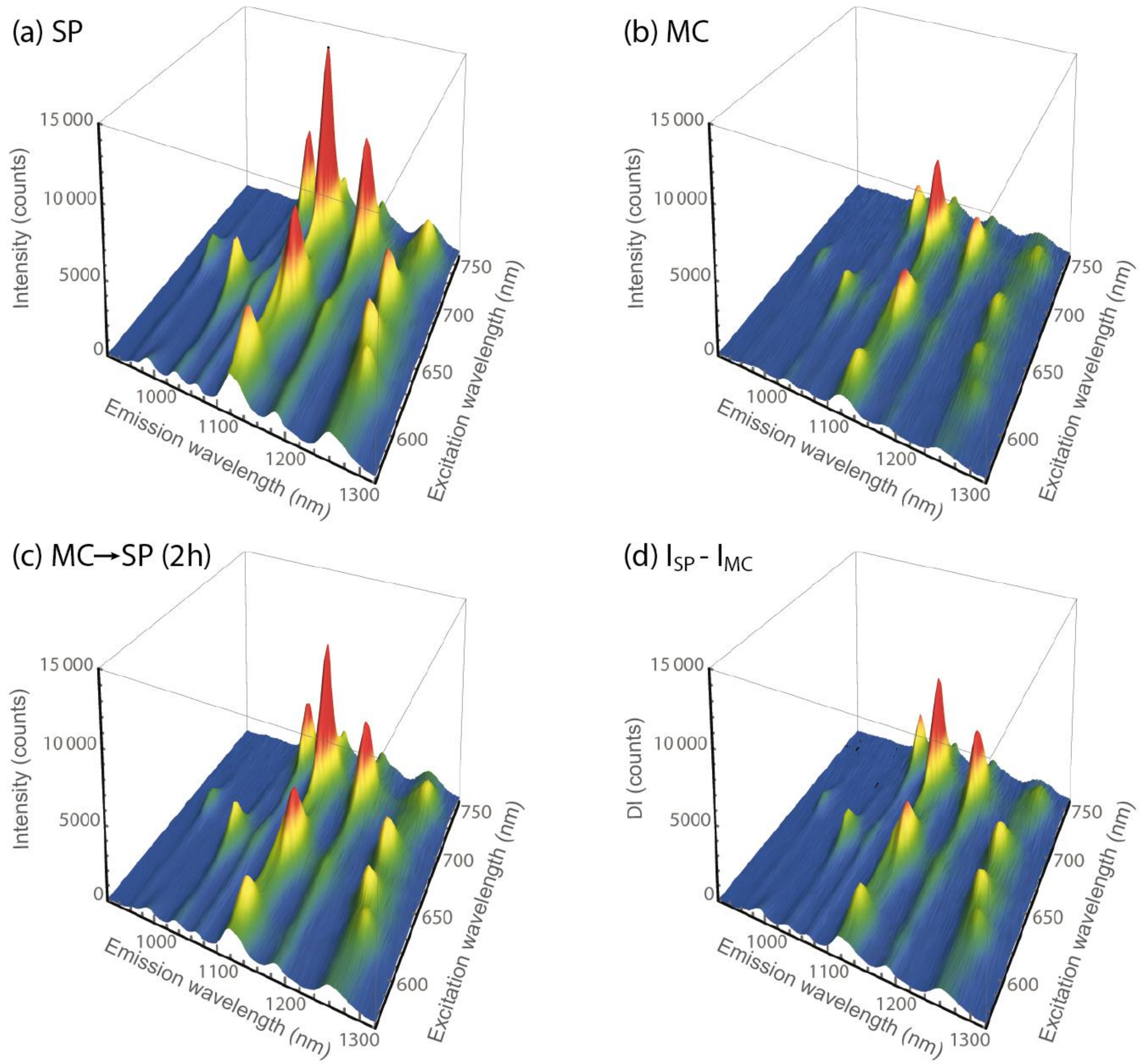

Supplementary Figure 31. Excitation-emission maps of the (a) SP-SWNTs, (b) MC-SWNTs after 1 h of UV irradiation at 367 nm and (c) mixed MC/SP-SWNTs sample, left in darkness for two hours. (24 h of darkness are required for complete back conversion to the SP-SWNT form). (d) PLE difference map obtained by subtracting the intensity of the SP-SWNT sample (a) from the MC-SWNT sample (b).

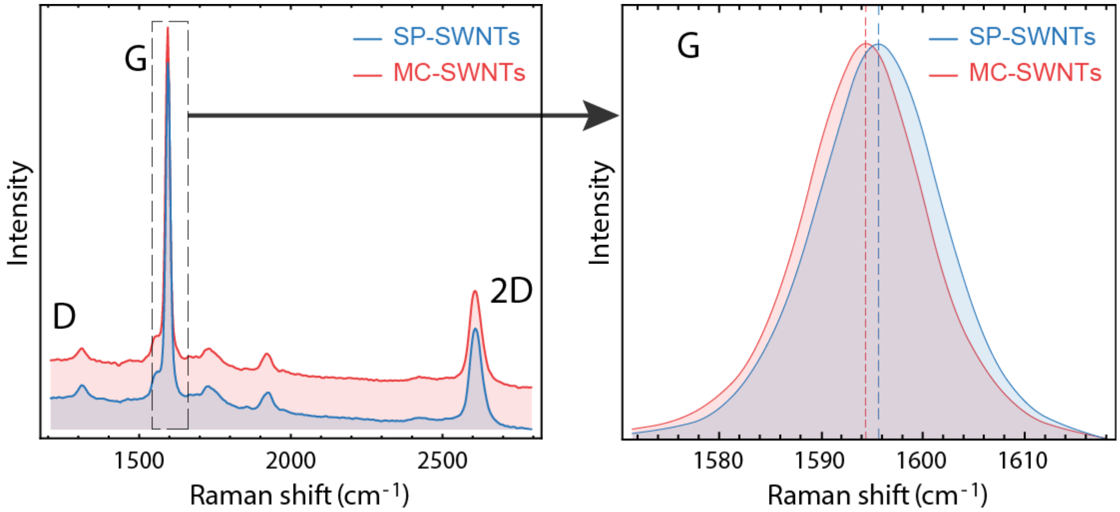

Supplementary Figure 32. (a) Comparison between the Raman signal of SP-SWNT and MC-SWNT, excitation wavelength 633 nm. (b) After SP-to-MC isomerization, a shift of the G band occurs. No appreciable shifts can be observed between the other bands.

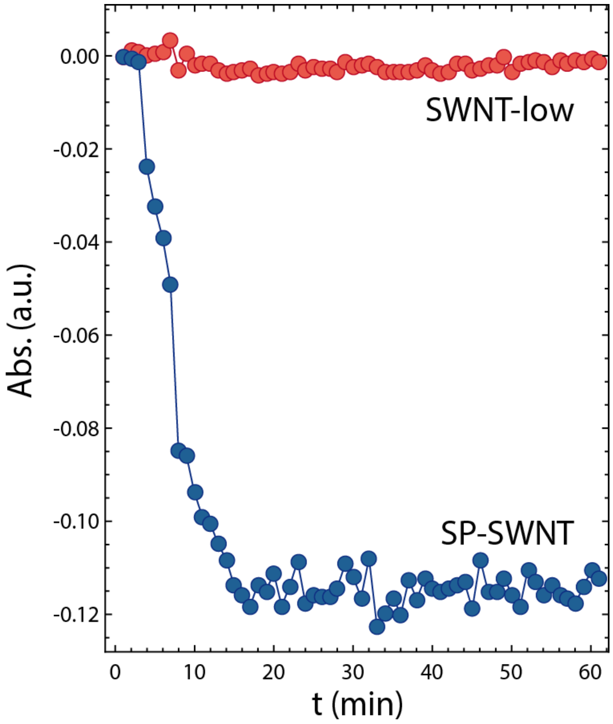

Supplementary Figure 33. Control experiment: Comparison of the intensity of the absorbance at 300 nm of the SWNT-low and SP-SWNT samples under UV light irradiation

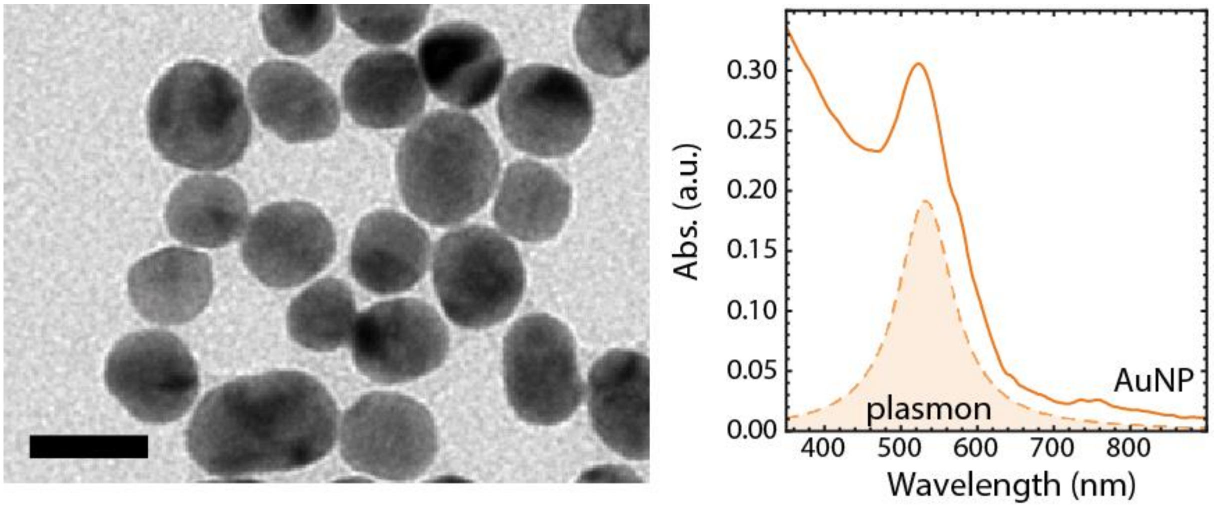

Supplementary Figure 34: TEM micrograph and absorbance of our AuNPs (scale bar 20 nm).

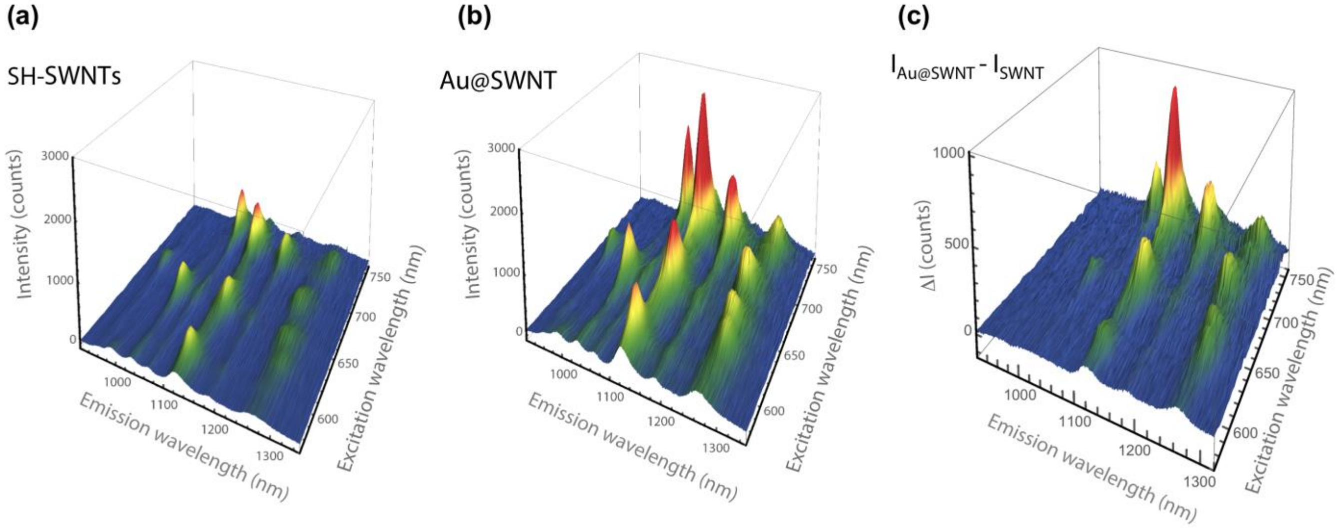

Supplementary Figure 35: Excitation-emission maps of the thiol-functionalized SH-SWNT (a) and Au@SWNT (b). The PLE difference map obtained by subtracting the intensity of the reference SWNT sample from the hybrid Au@SWNT sample is shown in panel (c).

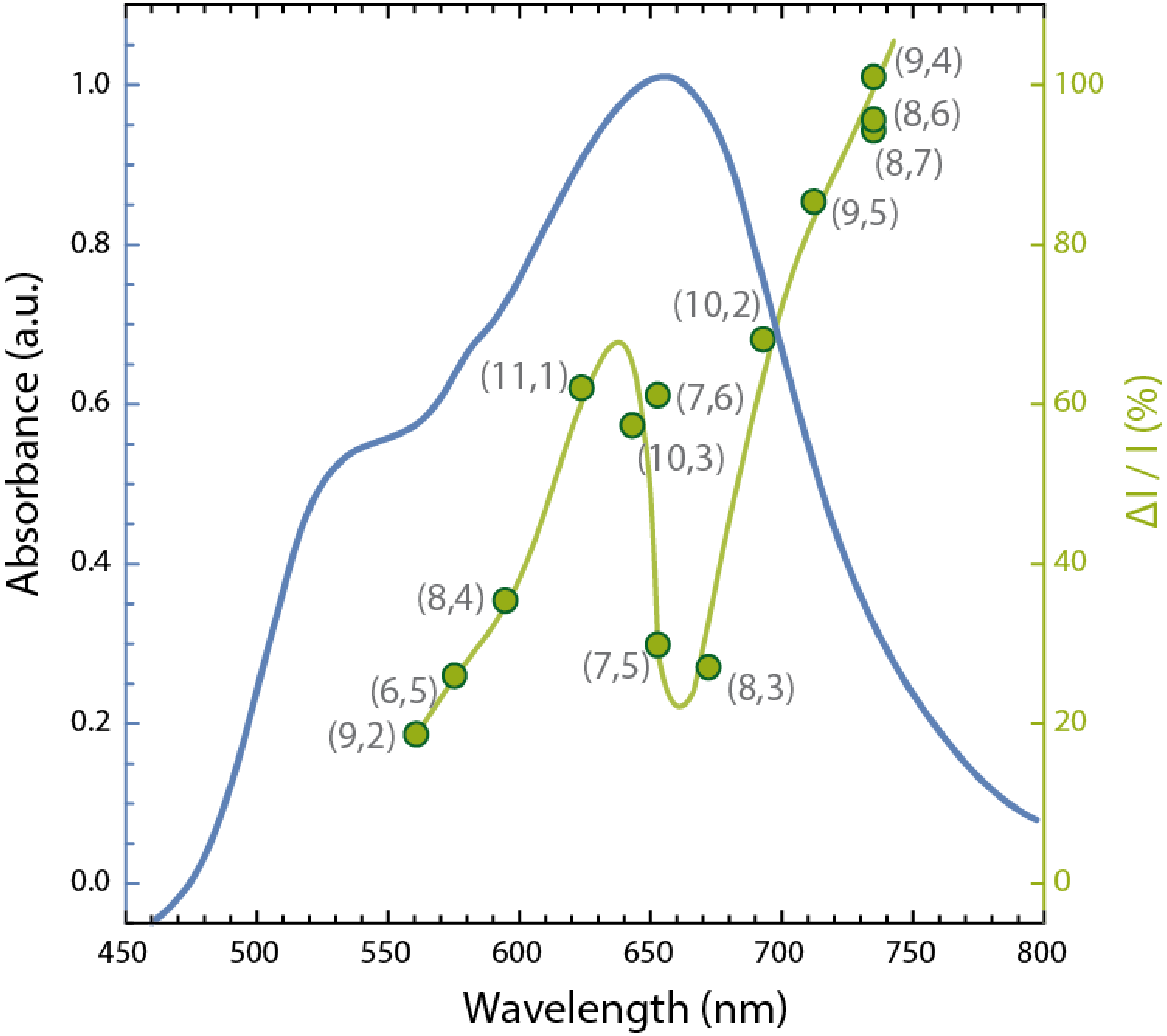

Supplementary Figure 36: Comparison between the absorption spectrum (blue curve) and the relative intensity increase of various Au@SWNT hybrid species (green dots, the line is a guide to the eye).

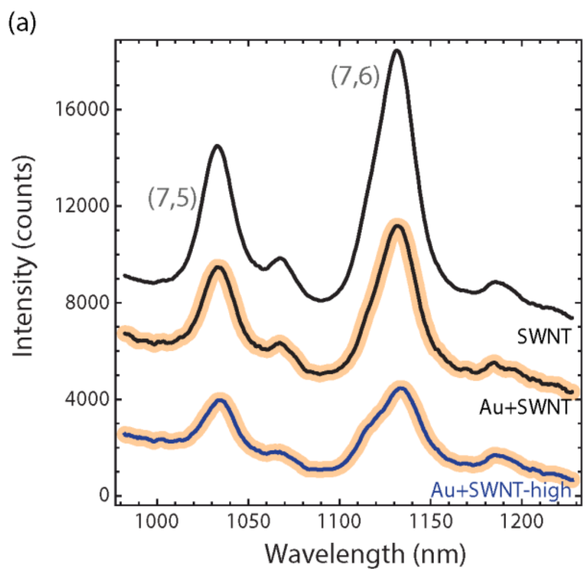

Supplementary Figure 37. Comparison between the emission spectra of the (7,5) and (7,6) (panel a) and the (10,2), (9,4), and (8,6) (panel b) nanotube species from: Pristine nanotubes (SWNT), pristine tubes mixed to AuNPs (Au+SWNT), and triazine-functionalized mixed with AuNPs (Au+SWNT-high).

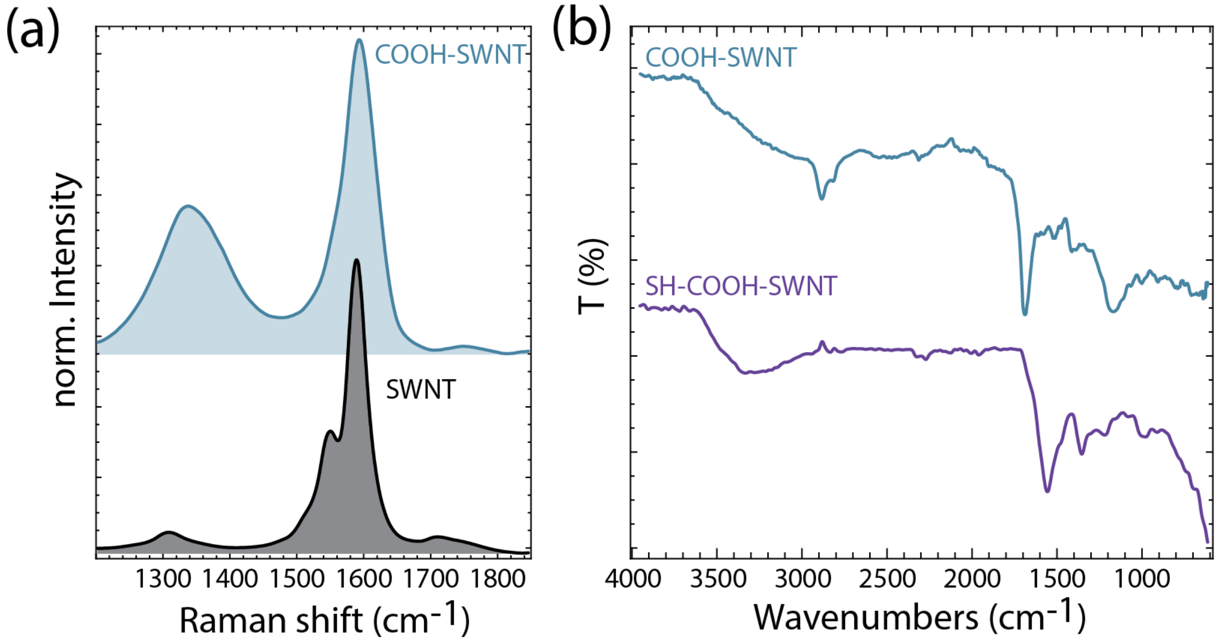

Supplementary Figure 38. (a) Comparison between the D and G lines of pristine (SWNT) and carboxylated (COOH-SWNT) nanotubes. (b) Comparison between the IR spectra of COOH-SWNT and SH-COOH-SWNT.

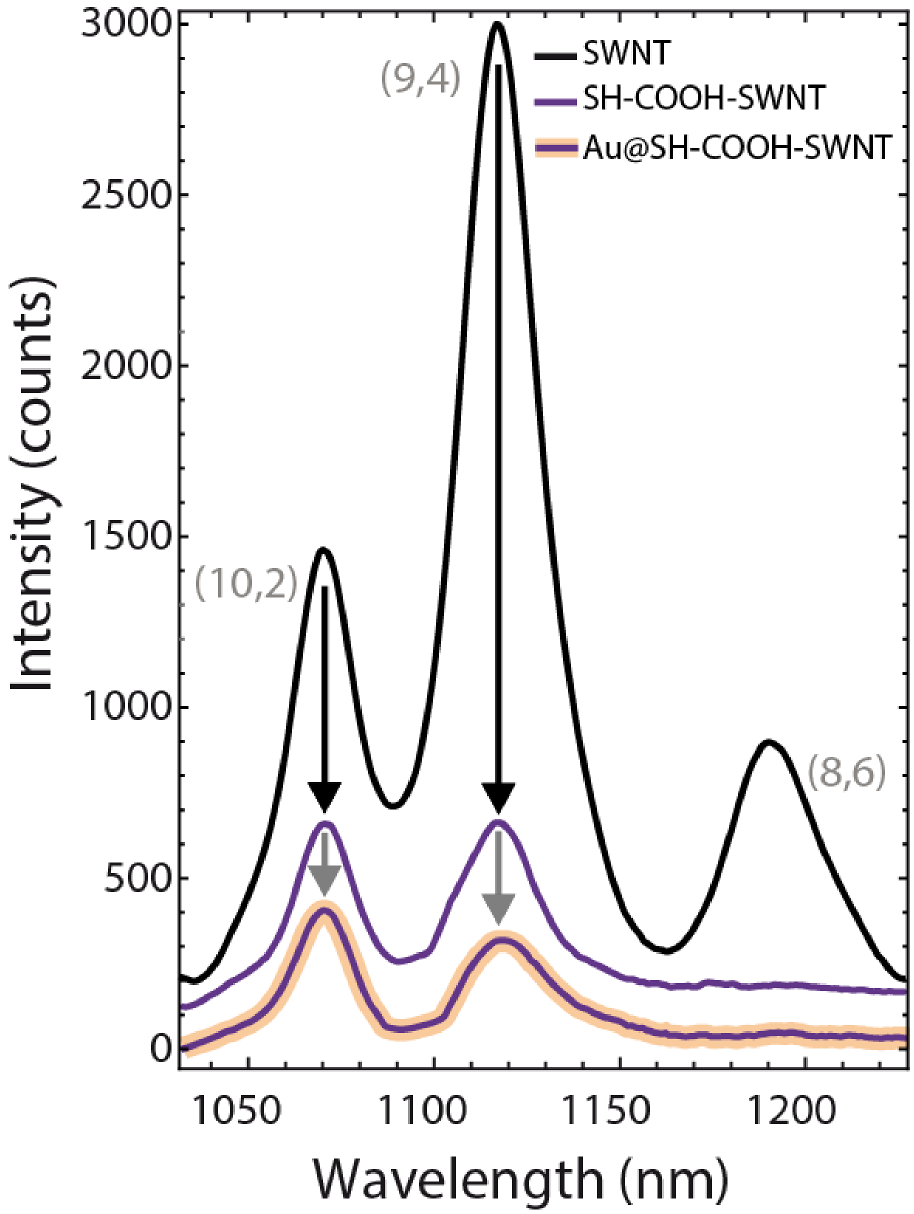

Supplementary Figure 39. Comparison between the emission of thiol-terminated carboxylated nanotubes before (SH-COOH-SWNTs) and after (Au@SH-COOH-SWNTs) AuNPs attachment.

Supplementary Tables

Supplementary Table 1. Results of the elemental analysis for 2-azido-4,6-dichloro-1,3,5-triazine.

Element	Experimental	Calculated
**C%**	19.1	18.8
**H%**	0.8	0
**N%**	45.1	44.0

Supplementary Table 2. Elemental analysis results for 1-(4,6-dichloro-1,3,5-triazine)-2,3,3-trimethylindolinium chloride.

Element	Experimental	Calculated
**C%**	16.4	16.0
**H%**	3.8	3.8
**N%**	48.7	48.9

Supplementary Table 3. Elemental analysis results for (4,6-diphenoxy-1,3,5-triazin-2-yl)-L-cysteine.

Element	Experimental	Calculated
**C%**	57.2	56.2
**H%**	4.5	4.2
**N%**	13.9	14.5

Supplementary Table 4. Summary of the elemental analysis for functionalized SWNTs: Fraction of N, C, H, S, density of functional groups, and fraction of SWNTs with respect to the total product. The element composition does not sum up to 100%, due to the metal catalyst and the chlorine not reported in here.

Sample	%N	%C	%H	%S	DFG	SWNT %
**SWNT**	0.1	81.1	0.8	0	0	100%
**SWNT-low**	4.2	70.8	1.9	0	1/100	88%
**SWNT-high**	12.5	68.2	2.0	0	1/25	64%
**SWNT-indole**	4.9	79.6	1.8	0	1/95	80%
**SP-SWNT**	5.1	74.1	2.1	0	1/100	73%
**SH-SWNT**	6.5	76.1	2.1	1.48	1/80	74%

Supplementary Table 5 Quantification of the SWNT functionalization by XPS. Summary of the functionalization ratio (FR) and density of functional groups (DFG) with respect to the SWNTs and the observed shift of the C 1s XP spectra. Additionally also the FRs of the triazine groups with respect to their chlorine and spiropyran content are given. The two samples denoted with a (*) were made with the same methods but based on SWNTs from different batches and processes (CoMoCAT® nanotubes).

Sample	FR_{XPS}(tri) (%; ±0.5%)	DFG_{XPS}(tri)	C 1s XPS shift (meV; ±37 meV)
SWNT	0	0	0
SWNT-low	1.0	1/104	119
SP-SWNT	0.9	1/108	112
SWNT-high	4.1	1/24	202
SWNT(*)-low	0.5	1/205	58
SWNT(*)-high	5.6	1/18	235

Supplementary Table 6. Results of the Raman characterization of the metallic tubes.

Sample	$\|E_F$ shift$\|$ (meV)	G^- Position (cm^{-1})	G^- FWHM (cm^{-1})
**SWNT**	70	1536	71
**SWNT-low**	40	1524	84
**SWNT-high**	20	1522	100
**SP-SWNT**	40	1528	85

Supplementary Table 7. Summary of the elemental analysis for COOH-SWNTs and SH-COOH-SWNTs: Fractions of N, C, H, S.

Sample	%N	%C	%H	%S
**COOH-SWNT**	0.9	62.5	2.4	0.032
**SH-COOH-SWNT**	3.8	49.1	2.8	10.4

Supplementary Note 1: Synthesis and characterization of the functionalized tubes

In this supplementary note we characterize our functionalization products. The intermediate azide and the final product are examined by thermogravimetric analysis (TGA), nuclear magnetic resonance (NMR), elemental analysis (EA), and infrared spectroscopy (IR). Raman spectroscopy identifies the nanotube species. High-resolution scanning transmission electron spectroscopy, coupled with local electron energy loss spectroscopy, allows to locally detect the presence of the functionality onto the nanotube sidewalls. 2-D excitation-emission spectroscopy proofs that the functionalized nanotubes emit light.

Schematic summary of the reaction steps

The synthetic steps towards the functionalized SWNTs are described in the methods section of the main manuscript text and schematically summarized in Supplementary Figure 1.

Characterization of the 2-azido-4,6-dichloro-1,3,5-triazine intermediate

The synthesis of 2-azido-4,6-dichloro-1,3,5-triazine through the reaction between sodium azide and 2,4,6-trichloro-1,3,5-triazine at low temperature was reported previously. [1] This compound is an interesting precursor for highly reactive nitrene intermediates. [2] To prove the successful creation of the product of reaction of sodium azide with 2,4,6-trichloro-1,3,5-triazine (Supplementary Figure 2a), we purified and characterized the intermediate azide at 0ºC. Supplementary Figure 2b shows the TGA thermograms of 2,4,6-trichloro-1,3,5-triazine and 2-azido-4,6-dichloro-1,3,5-triazine. The substitution of one of the chlorine atoms of cyanuric chloride dramatically changes the thermal behaviour. While 2-azido-4,6-dichloro-1,3,5-triazine starts losing weight at low temperature (purple line), its main decomposition temperature is

higher (ca. 200°C) than 2,4,6-trichloro-1,3,5-triazine (blue line). Elemental analysis (Supplementary Table 1), ^{13}C NMR (Supplementary Figure 3) and IR spectroscopy (Supplementary Figure 4) prove that the intermediate of the functionalization of SWNTs is 2-azido-4,6-dichloro-1,3,5-triazine.

In the ^{13}C NMR spectrum of 2-azido-4,6-dichloro-1,3,5-triazine, the two signals at 171 ppm and 172 ppm are assigned to the two types of carbon atoms of the compound as sketched in Supplementary Figure 3.

Supplementary Figure 4 shows the IR spectra of 2,4,6-trichloro-1,3,5-triazine (blue) and 2-azido-4,6-dichloro-1,3,5-triazine (purple). The appearance of the strong absorbance band at 2133 cm^{-1} in the IR spectra of 2-azido-4,6-dichloro-1,3,5-triazine due to the azide group proves the substitution of one of the chlorine of the cyanuric chloride by azide.

Comparison between covalently and non-covalently triazine-functionalized SWNTs

To prove the effective covalent attachment of the triazine onto the SWNTs, we performed a blank reaction designed to prevent the covalent attachment of the triazine onto the SWNTs. We followed the synthetic steps of the original reaction but without adding the sodium azide and thus without converting the 2,4,6-1,3,5-trichloro-triazine into 2-azido-4,6-dichloro-1,3,5-triazine. In this way the 2,4,6-1,3,5-trichloro-triazine can only non-covalently attach to the SWNTs.

For performing the blank reaction, SWNTs (1 g) were added to N-methyl-2-pyrrolidone (150 ml) and sonicated for 1 h. The mixture was then stirred at room temperature for one additional hour. 2,4,6-1,3,5-trichloro-triazine (1 g) dissolved in N-methyl-2-pyrrolidone (50 ml) was added at 0°C and stirred for 20 min. The temperature was raised to 25°C and it was stirred for 1 h. Finally, we left the mixture stirring at 70°C overnight. To compare the TGA response of covalently and non-covalently functionalized SWNTs, we evaporated the solvent of the blank

mixture at 70°C under vacuum. The results of the TGA analysis are shown in Supplementary Supplementary Figure 5. The TGA thermogram of the noncovalent sample is completely different from the covalently functionalized one. We observe (gray line in Supplementary Figure 5) two distinct onsets of decomposition at 140 and 440°C, which are due to the triazine and to the SWNTs. Our blank mixture consists of two separate compounds. The TGA of the SWNT-high (blue line in Supplementary Figure 5) does not show such an onset for the weight loss. The decomposition starts below 100°C and continues up to 430°C. This is assigned to the loss of trapped water molecules (low T), followed by the detachment of triazine molecules from the SWNT and their decomposition. Such broad decomposition spectra for functionalized tubes are well-known and abundantly reported in the literature. [3-5]

Synthesis and characterization of 1-(4,6-dichloro-1,3,5-triazine)-2,3,3-trimethylindolinium chloride and spiropyran

The reaction of dichlorotriazine with dimethyl indoline was performed for the first time in our experiments. Details of the synthesis are reported in the methods section of the main manuscript text. The results of the elemental analysis (reported in Supplementary Table 2) of the 1-(4,6-dichloro-1,3,5-triazine)-2,3,3-trimethylindolinium chloride are very close to the calculated contents, confirming the successful synthesis of the compound.

^{1}H NMR ($CDCl_3$): δ 8.25, 7.15-7.35 (aromatic segment), 1.25, 1.4, and 1.6 ($-CH_3$).

^{13}C NMR ($CDCl_3$): δ 170, 168, and 161 (triazine segment), 140, 136.9, 127.7, 125.6, 122.2, 117.7, 100 (aromatic segment and C=N), 26.4, 22.1, 20.2 (CH_3).

FTIR: 2890 (ν_{CH}), 1612 ($\nu_{C=C}$), 1557 ($\nu_{C=N}$) cm^{-1}.

In the IR spectra of 1-(4,6-dichloro-1,3,5-triazine)-2,3,3-trimethylindolinium chloride (see Supplementary Figure 6), the absorbance bands at 1612 and 1557 cm^{-1} are assigned to C=C and C=N bonds, respectively. The absorbance band of the aliphatic C-H bond is observed at 2890 cm^{-1}. In the ^{1}H NMR spectra of 1-(4,6-dichloro-1,3,5-triazine)-2,3,3-trimethylindolinium chloride (Supplementary Figure 7), the signal at 8.25 ppm is assigned to the proton 1, while the multiple signals at 7.15-7.35 ppm correspond to the protons 2, 3 and 4. Signals from the methyl groups are detected at 1.25, 1.4, and 1.6 ppm. In the ^{13}C NMR spectra of the compound (Supplementary Figure 8), signals at 170, 168, and 161 ppm are assigned to the carbon atoms of the triazine ring. Moreover, six signals from the aromatic ring as well as from the carbon of C=N bond are seen in the 100-140 ppm region. Three additional signals from the methyl carbons occur in the 20-27 ppm region.

Synthesis of 6-(3,3-dimethyl-2-methyleneindolin-1-yl)-1,3,5-triazine-2,4-diol

A solution of sodium hydroxide (0.2 g, 5×10^{-3} mol) in water (5 ml) was added to a degassed suspension of 1-(4,6-dichloro-1,3,5-triazine)-2,3,3-trimethylindolinium chloride (0.14 g, 4.1×10^{-4} mol) in water (30 ml) and then stirred at 80 °C for 15 min. The reaction was then cooled down to room temperature and diethyl ether (30 ml) was added to the solution. The organic phase was separated and the product was extracted from water by dichloromethane (4 × 20 ml). Sodium sulfate (1 g) was added to the organic phase and stirred for 1 h. After filtration the solvent was evaporated. The product was used for the next reaction without further purification.

Synthesis of (R)-6-(3',3'-dimethyl-6-nitrospiro[chromene-2,2'-indolin]-1'-yl)-1,3,5-triazine-2,4-diol

6-(3,3-dimethyl-2-methyleneindolin-1-yl)-1,3,5-triazine-2,4-diol (0.04 g, 1.48 × 10^{-4} mol) was dissolved in dry ethanol (15 ml) and degassed. 5-nitrosalicylaldehyde (0.037 g, 2.22 × 10^{-4} mol) was added to the solution and the mixture was sonicated at 25°C with 35 kHz for 2 h. The solvent was evaporated and the residue was dried under vacuum and purified by column chromatography using dichloromethane/methanol (50/2) as eluent.

^{1}H NMR ($CDC1_3$): δ 11.6 (-OH), 8.4, 8.1, 7.2, 7.1, 6.9 (aromatic segments), 5.6, 5.5 (vinylic segment), 1.3-1.2 ($-CH_3$).

^{13}C NMR ($CDC1_3$): δ 166.1, 161.6 (triazine segment), 140.5, 131.8, 129.8, 126.4, 124.9, 121.5, 119.4, 119.1117.4 (aromatic segments), 103.1, 102 (vinylic segment).

MS (ESI-TOF): calcd. for $C_{21}H_{17}N_5O_5$ 420.1263 $[M+H]^+$, found 420.1334 $[M+H]^+$

FTIR: 2920 (ν_{CH3}), 1734 ($\nu_{C=O}$), 1670 ($\nu_{C=C}$), 1525 ($\nu_{C=N}$), 1457-1341 (ν_{NO2}) cm^{-1}.

The compound was characterized by mass spectroscopy, ^{13}C NMR, and IR spectroscopy.

In the mass spectra, a peak at m/z = 420 proves that (R)-6-(3',3'-dimethyl-6-nitrospiro[chromene-2,2'-indolin]-1'-yl)-1,3,5-triazine-2,4-diol is successfully synthesized.

After the condensation reaction between 5-nitrosalicylaldehyde and 6-(3,3-dimethyl-2-methyleneindolin-1-yl)-1,3,5-triazine-2,4-diol, two new signals appear in the ^{13}C NMR spectrum of the product at 102 ppm and 103 ppm, Supplementary Figure 9(a). These signals are assigned to the carbon atoms of the vinylic bond of (R)-6-(3',3'-dimethyl-6-nitrospiro[chromene-2,2'-indolin]-1'-yl)-1,3,5-triazine-2,4-diol [Supplementary Figure 9(b)].

In the IR spectra of (R)-6-(3',3'-dimethyl-6-nitrospiro[chromene-2,2'-indolin]-1'-yl)-1,3,5-triazine-2,4-diol, the absorbance bands at 2920 cm^{-1}, 1734 cm^{-1}, 1670 cm^{-1}, 1525 cm^{-1}, and 1457-1341cm^{-1} are assigned to the methyl groups, tautomeric of hydroxyl groups to carbonyl, C=C

bond of aromatic ring, C=N bond of triazine ring and nitro group, respectively (Supplementary Figure 10).

<u>*Characterization of the reaction of dichlorotriazine with cystein*</u>

Synthesis of 2-chloro-4,6-diphenoxy-1,3,5-triazine

2-chloro-4,6-diphenoxy-1,3,5-triazine was synthesized and purified according to the reported procedure [6]. Briefly, a solution of phenol (1 g, 1.05×10^{-2} mol) and sodium hydroxide (0.42 g, 1.05×10^{-2} mol) in 10 ml water was added to a solution of cyanuric chloride (0.96, 5.2 10^{-3} mol) in dichloromethane (40 ml), dropwise at 0 °C. The mixture was stirred at room temperature for 1 hour and then refluxed for additional 6 hours. After that, the reaction was cooled down and filtered and the solvent evaporated. The crude product was dissolved in dichloromethane (5 ml) and then filtered. The product was precipitated in methanol, yield 70%.

^{1}H NMR ($CDC1_3$): a) δ 7.10-7.40 (phenoxy segments)

^{13}C NMR ($DMSO_{d6}$): δ 173.7, 172.4, (triazine segment), 151.2, 129.7, 126.7, 121 (phenoxy segments).

FTIR: 1557 ($\nu_{C=C}$, $\nu_{C=N}$), 1261 (ν_{C-O}) cm^{-1}.

Synthesis of (4,6-diphenoxy-1,3,5-triazin-2-yl)-L-cysteine

2-chloro-4,6-diphenoxy-1,3,5-triazine (0.1 g, 3.34×10^{-4} mol) was dissolved in NMP (15 ml) and triethylamine (0.05 ml, 3.5×10^{-4} mol). Cysteine (0.04 g, 3.3×10^{-4} mol) was added to the solution and the mixture was stirred at room temperature for 10 min. The temperature of the reaction was then raised to 70 °C and stirred for 24 h. After that, the mixture was cooled down and the solvent was evaporated under vacuum. The crude product was dissolved in water (20 ml) and extracted by dichloromethane (3 × 15 ml). Yield 45%.

^{1}H NMR ($DMSO_{d6}$): δ 7.1-7.4 (phenoxy segments), 6.7 (-NH-), 3.25-3.35 ($-CH_2$), 2.1-2.2 (-CH).
^{13}C NMR ($DMSO_{d6}$): δ 174, 166 (triazine segment), 172.2 (C=O), 152.2 130, 126, 122.4, (phenoxy segments).
MS (ESI-TOF): calcd. for $C_{18}H_{16}N_4O_4S$ 384.0892 $[M+H]^+$, found 384.3532 $[M+H]^+$.
FTIR: 3334 (ν_{OH}), 2863 (ν_{CH}), 2375 (ν_{SH}), 1731 ($\nu_{C=O}$), 1612 ($\nu_{C=C,}$ $\nu_{C=N}$) cm^{-1}.

After conjugation of cysteine to the 2-chloro-4,6-diphenoxy-1,3,5-triazine, a new signal in the ^{1}H NMR spectra of the product appeared at 6.7 ppm, see Supplementary Figure 11. This signal is assigned to the proton of the amine group which is conjugated to the triazine ring. This result proves that the nucleophilic reaction between the triazine ring and cysteine occurs mostly through the amino group of cysteine.

In the IR spectra of (4,6-diphenoxy-1,3,5-triazin-2-yl)-L-cysteine (Supplementary Figure 12) the absorbance band at 2375 cm^{-1} is assigned to the thiol group. This result proves that cysteine is attached to the triazine ring through amino functional group and that the thiol group remains unreacted.

In the mass spectra, the peak at m/z = 384.3 proves the successful synthesis of (4,6-diphenoxy-1,3,5-triazin-2-yl)-L-cysteine. The results of the elemental analysis (Supplementary Table 3) are consistent with the ^{1}H NMR (Supplementary Figure 11) and the mass spectra, proving the successful reaction between cysteine and 2-chloro-4,6-diphenoxy-1,3,5-triazine.

Raman Scattering

Supplementary Figure 13 depicts the Raman spectra of the radial breathing modes (RBM) of pristine (SWNT) and triazine functionalized (SWNT-low and SWNT-high) nanotubes excited at

633 nm. The lines of the (7,6), (8,4), (7,5), and (8,3) nanotube species are observed at these excitation energies. [7] No change in the RBM is observed for the different degrees of functionalization. The D and G bands of the Raman spectra are reported in Fig. 2c of the main text.

HR(S)TEM and EELS studies

Transmission electron microscopy (TEM) and spatially-resolved electron energy loss spectroscopy (SR-EELS) studies were performed on the functionalized nanotubes. Supplementary Figure 14a is a high resolution (HRTEM) micrograph where a bundle of single-walled nanotubes is visible. Some moieties are observed at the surface of these SWNTs. In order to locally analyse these objects, having access to chemical information, we performed spatially-resolved EELS (SR-EELS) measurements. This is a powerful tool to investigate such nanomaterials at

(sub-) nanometer scale [8-15].

We acquired a spectrum-image (SPIM) in the entire area shown in the high angle annular dark field (HAADF) image (Supplementary Figure 14b). Supplementary Figure 14d shows three EEL spectra extracted in the different areas marked in Supplementary Figure 14b. Each of these EEL spectra corresponds to the sum of nine spectra (3x3 probe positions of the SPIM). The carbon K edge at ~285 eV is present in all these EEL spectra. This C-K edge shows the characteristic peaks of sp^2 materials at 285 eV and 292 eV, corresponding to the π^* and σ^* contributions, respectively [8-11, 15]. The C-K edge displayed in Supplementary Figure 14d-(ii) is slightly different to the other two. Indeed, we observed that the fine structures near the edge (ELNES) of this spectrum indicate that we are dealing with a material less graphitic than those ones recorded in the other two regions (clean individual SWNT (iii) or a clean region of the bundle). Nitrogen

is also detected, see Supplementary Figure 14d-(ii). The ELNES analysis of this N-K edge indicates that this nitrogen corresponds to a pyrrolic-like atomic configuration [8-13]. Thus, the presence of this nitrogen, its atomic configuration, and the atomic configuration of the associated carbon, reveal that this nitrogen is associated to the presence of triazine.

Furthermore, it is also confirmed by the fact that these molecules are the only source of nitrogen in the samples. From the local TEM analyses, we conclude that the observed moieties correspond to the triazine functionalized molecules localized at the surface of the SWNTs.

Absorption and 2D emission spectroscopy of the SWNTs-low and SWNTs-high

Supplementary Figure 15a-c shows the full 2D luminescence map of pristine SWNT, SWNT-low and SWNT-high (cp. Fig. 2). Chiral species are labelled with the (*n*,*m*) indices.[8,9,16] Supplementary Figure 15d shows their absorption spectra.

Supplementary Note 2: Computational studies

The structure of triazine-functionalized carbon nanotubes were calculated for armchair and zig-zag nanotubes. For this paper we concentrate on the exemplary (8,0) nanotube: Calculations were performed on non-periodic structures in which dangling bonds at both ends were terminated by hydrogen bonds (Supplementary Figure 16). The convergence thresholds for energy change, maximum force, and maximum displacement between optimization cycles were set at $2.0 \cdot 10^{-7}$ kcal·mol, 0.01 kcal/mol/Å and 0.0001 Å respectively. The optimization stopped when the energy convergence was satisfied along with either the displacement or gradient criterion. The default optimization algorithm (EF) was used. The NEB calculation were performed using the ASE package. [17].

We calculated the reaction pathway for the covalent attachment of triazene onto the nanotube using climbing image nudged elastic band (CI-NEB) method [46] with the ASE package.[17] It constructs a series of atomic configurations along a minimum energy path (MEP), which follows atomic configurations corresponding to neighboring local minima. The CI-NEB showed that the MEP for the cycloaddition of the nitrene on the (8,0) nanotube lacks an activation barrier and the reaction of a single triazene with the clean SWNT occurs without an evident transition state. Initial and final structures were optimized and characterized by means of a force calculation in order to verify that two minima structures are used. The MEP was delimited by 42 intermediate configuration. Supplementary Figure 17 was calculated considering an initial separation of ca. 6 Å between the barycenters of the two endpoints. The forces on all atoms in each image of the CI-NEB chain were converged to 0.05 eV.

The nanotube orbitals of the functionalized (8,0) tubes showed the characteristic π-orbitals structure of semiconducting nanotubes. [16] Particularly impressive is the minimum amount of

distortion by the triazine in Supplementary Figure 18a (isosurface at $7.6 \cdot 10^{-3}$ e^{-} nm^{-3}). The HOMO (π-character) contains contributions from the nanotube and the triazine as visible for the isosurface in Supplementary Figure 18b (isosurface at $2.5 \cdot 10^{-3}$ e^{-} nm^{-3}).

Supplementary Note 3: Quantification of the functionalization product

In this Supplementary Note we characterize our functionalized carbon nanotubes and compare the results from different experimental methods. Elemental analysis, thermogravimetry, and X-ray photoelectron spectroscopy are used to prove the efficient attachment of the triazine groups onto the SWNTs as well as to quantify the amount of functional groups conjugated onto the tubes. Infrared absorption spectroscopy confirms the presence of the additional vibrational modes of the groups attached to the tubes.

Calculation of the density of functional groups and of the carbon nanotubes fraction

Single walled carbon nanotubes with different but well-defined numbers of functional groups were synthesized by in situ [2+1] cycloaddition reaction with azidodichloro-triazine (compound **1**) at ambient conditions. An estimation of the amount of functional groups conjugated onto the SWNTs is obtained by evaluating the density of functional groups (DFG), defined as the number of triazine rings per number of carbon atoms of the SWNT. The DFG from elemental analysis (EA) is given by the ratio between the total mass of N atoms within the functional group divided by the total nitrogen content of the product. The results of the elemental analysis, the estimated DFG, and the density of SWNTs are summarized in Supplementary Table 4 for various samples.

TGA analysis

Thermogravimetric analysis (TGA) showed that the amount of SWNT was reduced by 12% and 36% due to the triazine functional groups of the SWNT-high and SWNT-low samples, respectively (Supplementary Figure 19a). Based on these mass ratios, the DFG of SWNT-high was 1/25 and of SWNT-low was 1/100. These results are in excellent agreement with the elemental analysis results.

Each reaction was repeated at least ten times for evaluating the reproducibility of the functionalization. The nitrogen contents slightly changes from batch to batch, with a maximum deviation from the mean value of 1% (SWNT-low) and 1.3% (of SWNT-high), respectively (Supplementary Figure 19b). The nitrogen content is an indicator for DFG, proving that our approach produced SWNT with a well-defined amount of functionalities.

IR spectroscopy

In the IR spectra, characteristic bands of aziridine rings usually appear in the 1000-1250 cm^{-1} region and are assigned to symmetric aziridine ring breathing modes and to the C-N bonds in which carbon is sp^3 hybridized (sp^3C-N bonds) [18]. Instead of those peaks, new absorbance bands, not present in pristine SWNT, arise at 1296 cm^{-1} and 1305 cm^{-1} for SWNT-low and SWNT-high, respectively (Supplementary Figure 20a-c). They are assigned to sp^2C-N bonds and indicate efficient attachment of triazine groups onto SWNTs through carbon atoms with sp^2 hybridization. In the IR spectrum of SWNT-indole (Supplementary Figure 20d), the absorbance bands at 1612, 1567 and 1297 cm^{-1} are assigned to the C=C, C=N and C-N bonds, respectively. The IR spectrum of SP-SWNT (Supplementary Figure 20e) exhibits absorbance bands at 2920, 1670, 1516, 1300, and 1150 cm^{-1}, that correspond to the C-H, C=C, C=N, C-N, and C-O bonds, respectively.

In the IR spectrum of SH-SWNT (Supplementary Figure 20f) the broad band in the 3300-2400 cm^{-1} region is due to the carboxyl functional group of cysteine. The band in the 1540-1680 cm^{-1} region corresponds to the absorbance of the carbonyl group of cysteine overlapped with the absorbance of C=C and C=N bonds of SWNT and triazine.

X-ray photoelectron spectroscopy (XPS)

Sample Composition

Supplementary Figure 21 shows survey XPS spectra of SWNT, SWNT-low, SP-SWNT, SWNT-high, and a gold reference that was handled the same way as the drop-coated samples. All spectra show Au features, in particular the spin-orbit-split 4f doublet at E_B = 83.96 and 87.64 eV as shown in the upper right inset. The full widths at half maximum (FWHM) of the respective peaks of 0.57 and 0.58 eV indicate an instrumental resolution of 380 meV. The Au reference shows negligible traces of adventitious carbon at E_B = 284.4 eV and no other contaminations. The SWNT samples show a large carbon (C 1s) signal around E_B = 284.4 eV and smaller peaks due to oxygen species (O 1s) around E_B = 532 eV. SWNT-low, SP-SWNT, and SWNT-high also contain nitrogen (N 1s, at ca. E_B = 400 eV) and chlorine[a] (Cl 2p, at ca. E_B = 201 eV), which proves the successful SWNT functionalization. The occurrence of oxygen in the SWNT samples is mainly due to their inherent oxidation at defect sites.

Carbon 1s Spectra

Supplementary Figure 22 details the C 1s XP spectra of the pristine SWNT and the highly functionalized SWNT-high. The spectrum of SWNT is clearly dominated (52.4% of the total area) by an asymmetric peak [19] with a maximum at E_B = 284.30 eV due to the graphitic sp^2-hybridized carbon backbone of the SWNT. In the long asymmetric tail on the higher-binding-energy side of the spectrum we allocated additional Voigt-shape peaks ascribed to sp^3-hybridized carbon (E_B = 284.70 eV, 17.9%), hydroxy (**C**-OH, E_B = 286.10 eV, 7.4%), carbonyl (**C**=O, E_B = 287.45 eV, 4.2%), and carboxyl groups (**C**OOH, E_B = 288.89 eV, 1.4%), and a π-π^* shake-up

[a] We found very small traces of chlorine in the pristine SWNT as well. They are most probably due to residues from molecular sieves added to the solvent we used for sample preparation.

process in the sp^2 carbons [20] (E_B = 290.27 eV, 16.7%) on top of a Tougaard background [21]. The sp^3 and carbon-oxygen species are due to defect sites in the SWNTs and their inherent oxidation. These constitute 30.9% of the total carbon content, leaving 69.1% of sp^2-hybridized carbon.

The inset graph of Supplementary Figure 22 shows the C 1s spectra of the functionalized samples. They closely resemble the spectra of the pristine SWNT but are energetically shifted. We fitted the spectrum of SWNT-high by combining the same peak-area ratios and peaks we used for SWNT, but shifted in E_B by +202 meV, with two additional Voigt peaks at E_B = 285.84 eV and 288.01 eV. The latter two peaks have a peak-area ratio of 3:2 and are assigned to the three carbon atoms of the triazine unit and the two carbon atoms of the SWNT to which the bridging nitrogen is attached (C_2 unit). The C 1s spectra of all other functionalized SWNTs were fitted by the identical procedure.

The appearance of the additional peaks for SWNT-high and SWNT-low proves the actual *covalent* functionalization. The large chemical shift in E_B by +3.5 eV of the peak ascribed to the attachment C_2 units is striking. It suggests that the functionalization did not end with the formation of an aziridine moiety but rather resulted in the local restoration of the SWNT's conjugated sp^2-hybridized carbon system by ring opening. The bridging nitrogen atom's electron lone pair than joins the conjugated π-electron system of the SWNT by mesomerism with the corresponding iminium-cation state, Supplementary Figure 23. The chemical shift observed in XPS agrees reasonably well with the calculated binding energy (4.2 eV, see main text). This increases the electron density in the SWNT and explains the strong shift of the peak we ascribed to the attachment C_2 unit. As illustrated by the inset graph of Supplementary Figure 22, the C 1s spectra of the samples shift as wholes according to the functionalization. The values of the shifts

are summarized in Supplementary Table 5. A discussion of the phenomenon is presented in Supplementary Note 4.

Quantification of the Covalent Triazine Functionalization

The functionalization ratios of triazine groups (FR_{XPS}(tri)) on the SWNTs were determined by relating the respective total triazine N 1s spectral area of each sample to its sp^2 C1s area (i.e., the sum of sp^2 and attachment C_2-unit peak areas). To this end measurement statistics, relative elemental sensitivity factors [22], and the stoichiometry were taken into account. We define FR_{XPS}(tri) as the percentage of functionalized sp^2-carbon C_2 units.[b] To compare the XPS results with those of the elemental analyses (EA) we also determined the respective densities of functional groups (DFGs), Supplementary Figure 24.[c] The results are summarized in Supplementary Table 5. While our EA determined the mass percentage (wt%) of the elements C, H, N, and S contained in our samples, XPS quantified the relative amounts of *all* constituting elements (C, N, O, and Cl) but H. Elemental analysis, TGA, and XPS excellently agree in the obtained density of functional groups.

Nitrogen 1s Spectra

The N 1s spectra shown in Figure 2 of the main text and depicted in Supplementary Figure 25 (normalized to the sp^2 carbon peak) were each decomposed into Voigt peaks on a Shirley-type

---

[b] $\mathrm{FR_{XPS}(tri)} = \frac{\mathrm{Area_{XPS}^{N\,1s}(tri)}}{\mathrm{Area_{XPS}^{C\,1s}(sp^2\;C_2\;units)}} \times 100\%$, with $\mathrm{Area_{XPS}^{i}(j)}$ as core level i XPS peak area of constituent j.

[c] $\mathrm{DFG_{XPS}(tri)} = \frac{\mathrm{Area_{XPS}^{N\,1s}(tri)}}{\mathrm{Area_{XPS}^{C\,1s}(\;sp^2\;atoms)}} = \frac{1}{2}\mathrm{FR_{XPS}(tri)}$; and $\mathrm{DFG_{EA}(tri)} = \frac{\frac{M_{\mathrm{N}}(\mathrm{tri})}{\mathrm{wt\%(N)}} - M(\mathrm{tri})}{M(\mathrm{C})}$, with M(i) as molar mass of compound i and M_{j}(i) as molar mass of compound i with respect to element j.

background [23]. SWNT-high and SWNT-low were fitted with two peaks having an area ratio of 1:3 that we assigned to the single bridging (peak B) and the three triazine nitrogen atoms (peak A). The total triazine N 1s peak areas were used to determine the FR_{XPS}(tri) and DFG_{XPS}(tri) values given in Supplementary Table 5.

In the N 1s spectrum of the spiropyran-functionalized SP-SWNT an additional signal at E_B = 405.49 eV (peak C) is clearly visible. We assigned it to the nitro group of the spiropyran molecule assembled at the triazine anchor group [24], thereby proving its successful synthesis. The N 1s signal between E_B = 397 and 402 eV for SP-SWNT is similar to that of SWNT-low. The additional intensity is due to the second nitrogen atom of the molecular switch (peak D).

We determined the FR_{XPS}(spiro) of the spiropyran groups with respect to the available triazine units by comparing the N 1s XPS peak areas. The result of 65% gives the average number of spiropyran units per triazine anchor as 1.3. Statistically 30% of the triazine units substituted both of their chlorine atoms with a spiropyran switch, while 70% carry a single switch. This proves the effectiveness of this preparation.

Chlorine 2p Spectra

Cl 2p spectra for SWNT, SWNT-low, SP-SWNT, and SWNT-high are depicted in Supplementary Figure 26. The finite background in the SWNT spectrum (black) hints at trace amounts of chlorine. It originates most likely from the molecular sieves added to the solvent used for sample preparation. We took the SWNT Cl 2p spectrum as background for all other Cl spectra, scaling it to match their respective secondary-electron signals. The subtracted spectra were decomposed into doublets with peak-area ratios of 2:1 and a spin-orbit splitting of 1.5 eV on top of linear backgrounds. The $2p_{3/2}$ components (peak K) were found at E_B = 200.48 eV for SWNT-high and SP-SWNT and at E_B = 200.01 eV for SWNT-low.

By relating the Cl 2p peak areas to the respective triazine N 1s peak areas we determined the number of Cl atoms per triazine unit and the FR_{XPS}(Cl) (in brackets). For SWNT-low it is ca. 2 (100%), for SP-SWNT ca. 0.7 (35%), and for SWNT-high ca. 0.5 (25%).

These numbers indicate the following. First, during the low-temperature triazine-functionalization reaction for SWNT-low (and SP-SWNT) no chlorine was lost. We covalently couple intact dichlorotriazine functional groups to SWNTs. Second, the assembly of the spiropyran via SWNT-indole did not lead to any additional losses of chlorine atoms apart from the ones substituted. In SP-SWNT all triazine units carry on average 1.3 spiropyran molecular switches and 0.7 Cl atoms. In other words, 30% of the triazine units are doubly substituted with spiropyran, 70% have a spiropyran and a Cl atom attached. This proves that the synthesis of the spiropyran at the SWNT actually proceeds quantitatively. It also shows that all triazine groups that are covalently coupled to an SWNT may be further functionalized effectively. Third, the triazine functionalization at 70°C for SWNT-high caused a large but not complete loss of 75% of the Cl atoms. [d] Optimized reaction conditions will find an ideal trade off between functionalization density and preserving triazine Cl content.

[d] When XPS measurements were repeated with samples, there was no indication of any losses of functional groups by X-ray beam damage (cf. the inset in Supplementary Figure 26).

Supplementary Note 4: Change in Fermi energy

In the previous Supplementary Notes we focused on the chemical and morphological properties of the functionalized products, demonstrating the presence of the covalently attached functional groups and quantifying the density of groups attached onto the nanotube sidewall. We have, moreover, shown the 2-D excitation-emission charts for the nanotubes with different degrees of coverage, proving that the conjugation of the functional groups preserves the π-conjugation of the carbon network. Even if the covalent functionalization of the SWNTs does not perturb their optoelectronic structure, it can still contribute with additional charges to the nanotubes' delocalized π-system. In this Supplementary Note we focus on the investigations of this charge-transfer effect/doping and its quantification. For a careful characterization, we resort to different experimental techniques. Supplementary Figure 27 summarizes their results: The Fermi level of the functionalized tubes shifts upwards when increasing the density of functional groups. The p-doping of the pristine SWNTs gets (Supplementary Figure 27, left) compensated by the functionalization, uplifting the Fermi energies of the functionalized tubes close to the neutral point for SWNT-low (middle) and above in SWNT-high (right). The C 1s levels in the XPS spectra are sensitive to the functionalities conjugated onto the tubes. Their binding energy shift provides a useful indicator of the Fermi energy shift induced by the covalent attachment of functional groups. Raman investigations on small-diameter metallic nanotubes complement the XPS findings. Beyond the effect induced by the covalent attachment of the functional groups, in the last part of this note we analyse the optically-triggered effects due to the spiropyran-to-merocyanine isomerization when SP-SWNT are exposed to UV radiation.

X-ray photoelectron spectroscopy

Our XPS study provided strong evidence for an electronic interaction between the triazine unit and the functionalized SWNT. The C 1s XP signal of a C_2 unit under the bridge to the triazine unit shifted by 3.5 eV to higher binding energy. A corresponding shift to lower binding energy was observed for the N 1s signal of the bridging nitrogen atom with respect to the nitrogen in the triazine. The large opposite chemical shifts suggest that the electron lone pair of the bridging nitrogen atom contributes to the conjugated π-electron system of the SWNT, see the scheme in Supplementary Figure 23. This likewise manifests by an increase in Fermi energy. As illustrated in Supplementary Figure 28 the shift of the C1s XP level to higher binding energy directly reflects an upward shift of the Fermi level towards the SWNT conduction-band. Interestingly, a deposition of triazine on graphene without chemical interaction results in the opposite behavior (lower C 1s binding energy and decrease in Fermi energy). [24]

The XPS peak shifts were independent of the actual amount of SWNT material covering the substrate. This implies the induction of a negligible photovoltage and/or a low-resistance junction between the Au substrate and the drop-coated SWNT film. Therefore the Fermi levels E_F of the electron analyzer and the sample were aligned in the XPS measurements and the observed shift of the C 1s peak position with increasing functionalization ratio directly reflected the shift of the SWNT Fermi level. E_F shifts upwards towards the conduction band of the semiconducting SWNT. When referenced to the analyzer Fermi level the C1s XPS signal must shift downwards towards higher binding energy E_B (see Supplementary Figure 28).

In Supplementary Figure 29 we plot FR_{XPS}(tri) as a function of the observed C 1s peak shift ΔE for the SWNTs. We also investigated two additional triazine-functionalized samples, prepared with the same procedures, one with less (SWNT$^{(*)}$-low) and one with more (SWNT$^{(*)}$-high)

functional groups based on SWNTs coming from different production methods and batches (CoMoCAT®) and referenced against their respective pristine SWNTs.

Raman characterization of the metallic tubes.

The Raman spectra confirm the functionalization of metallic nanotubes through changes in peak width. The G band of metallic carbon nanotubes consists of two bands, labelled as G^+ at ca. 1580 cm^{-1} due to the transverse optical phonon (TO), and G^- at ca. 1540 cm^{-1} due to the longitudinal optical phonon (LO). The LO mode responsible for the G^- band is very sensitive to shifts of the Fermi energy level and its broadening can be used to quantify the doping in metallic SWNTs [26]. We monitored the G band of metallic nanotubes with various functionalization levels under 532 nm laser excitation, where metallic tubes were resonantly excited. For each sample we acquired at the same spot the G and the radial-breathing mode (RBM) spectra to control the CNT chirality distribution at the analysed position (Supplementary Figure 30a,b). We identified the metallic (9,3), (8,5), and (7,7) species.

To calculate the Fermi energy shift, we used the model of the LO peak broadening discussed in Ref. [26]. A shift in the Fermi level away from its intrinsic position (crossing of valence and conduction band) continuously increases the LO phonon lifetime and decreases the full width at half maximum (FWHM) of the LO peak. As a rule of thumb one half of the maximum FWHM indicates the E_F at half the LO energy. The maximum FWHM value for the analysed species (d=0.85 nm) is 100 cm^{-1} and the phonon energy is 187 meV. [27] We considered a Lorentzian dispersion model for fitting our data (Supplementary Figure 30d), obtaining values of the doping levels of -70 meV for the pristine SWNT, -40 meV for SWNT-low and SP-SWNT, and +20 meV for SWNT-high. The positive sign of the E_F shift was obtained from the 6 cm^{-1} decrease in frequency of the 2D Raman mode between the pristine SWNT and SWNT-high. [26,28] It agrees

with the increase in Fermi energy obtained from the XPS data. Overall, ΔE_F from Raman is smaller than observed in XPS. This is explained by the limited number of small-diameter tubes accessed in Raman scattering, whereas XPS obtains a mean doping level across all nanotubes.

Effect of the SP-to-MC isomerization

Under UV irradiation, SP converts into MC. As a consequence of the SP-to-MC isomerization, the emission of the tubes gets quenched. In Supplementary Figure 31a we report the excitation-emission map (in 3-*D* to highlight the intensity changes) displaying all the nanotube species contained within the SP-SWNT sample. After the conversion, the emission is quenched in the MC-SWNT sample (Supplementary Figure 31b). Unlike free MC, MC-SWNT does not isomerize back to the SP-SWNT upon visible light irradiation. It thermally gets back to the SP-SWNT if left in darkness. Supplementary Figure 31c shows the recover of 75% of the emission intensity after 2 h of darkness. For a full recovery of the emission, the samples needed to remain in darkness for 24 h. The net effect of the SP-to-MC conversion is quenching of the SWNT emission, without any change in the spectral position of the emission bands (Supplementary Figure 31d). To quantify the amount of doping due to the isomerization, we focused on the Raman analysis of semiconducting SWNTs. Similarly to the metallic nanotubes, semiconducting nanotubes' G band consists of two sub-bands, labelled G^+ and G^- as well but arising from different modes than in metallic tubes. [26,27] For semiconducting SWNTs, the LO phonon contributes to the G^+ band (at 1590 cm^{-1}) while the TO contributes to the G^- band (at ca. 1565 cm^{-1}). Doping thus affects differently semiconducting SWNT than the metallic SWNT, shifting the position of the G^+ band. This can be used to quantify the shift of the Fermi energy [28]. The Raman spectra of SP-SWNT and MC-SWNT (obtained by UV light irradiation of the SP-SWNT to promote SP-to-MC conversion) are shown in Supplementary Figure 32. The only difference

between the two spectra is given by a shift of 2 cm^{-1} of the G^+ band position, suggesting an upshift of 0.2 eV of the Fermi energy from comparing with the result of Ref. [28].

Control experiment on the effect of UV irradiation

To confirm that the observed effects are due to the SP-to-MC conversion and rule out UV exposure damages, we exposed to UV irradiation two samples with similar characteristics but one with- (SP-SWNT) and the other without (SWNT-low) spiropyran. In Supplementary Figure 33 the temporal change of the absorbance at 300 nm is monitored. While the onset of the merocyanine absorption band can be observed in SP-SWNT, no changes occur in the SWNT-low sample, ruling out any damage by the UV light exposure.

Supplementary Note 5: Nanoplasmonic hybrids

In this Supplementary Note we report on the characteristics of the nanoplasmonic hybrids Au@SWNTs. Additionally, we discuss a set of control experiments for plasmonic enhancement in SWNTs.

Optical and morphological properties of the AuNPs

For the synthesis of the AuNPs, please refer to the Methods section of the main manuscript text. TEM micrographs of the AuNPs show a distribution of the particle size between 10 and 20 nm (Supplementary Figure 34). For such an ensemble absorption spectroscopy reveals the presence of the plasmon absorption band of the AuNPs peaked at 520 nm.

2D emission spectroscopy of the Au@SWNTs hybrids: Emission enhancement

Once coated with the AuNPs, we observe enhancement of the emission of the nanotubes. Supplementary Figure 35a reports the excitation-emission map (in 3-*D* to highlight the intensity changes) displaying all the nanotube species contained within the thiol-functionalized SH-SWNT sample. After the attachment of the gold nanoparticles, the emission of Au@SWNT is enhanced (Supplementary Figure 35b). The net effect of the interaction with the plasmonic particles is the enhancement of the emission of the different species, without any change in the spectral position of the emission bands (Supplementary Figure 35c).

The relative change of the emission intensity depends on the specific species and ranges between 20% and 100%. In Supplementary Figure 36 we plot the relative change of the emission intensity *vs.* excitation wavelength of the considered species (green dots, the curve is a guide to the eye) and compare it with the absorption spectrum of the Au@SWNT hybrids (blue curve).

Interestingly, there is a systematic red-shift between the plasmonic bands of the gold particles and the excitation resonance profile of the carbon nanotubes. The dependence of the enhancement on excitation wavelength with respect to the plasmonic response will be studied in a future paper.

Nanoplasmonic hybrids - control experiments: Role of the covalently-bound thiols and contribution of the triazine-based covalent attachment

To highlight the importance of the thiol-based attachment in our hybridization process, we performed two sets of control experiments: One control shows the relevance of the thiol-triazine based immobilization of the Au nanoparticles on the SWNTs and the other compares standard covalent functionalization with our cycloaddition-based method.

The first set of control samples were prepared following the very same procedure as for the Au@SWNTs hybrids but starting each time from different sets of tubes, one for each step of the synthesis leading to the SH-SWNTs. In Suppplementary Figures 37a,b we compare the emission of the pristine tubes (SWNT) with the emission of the hybrids obtained by mixing gold with pristine tubes (Au+SWNT) and tubes functionalized with triazine groups but without the thiol-termination (Au+SWNT-high). The emission from the hybrids is always weaker than the emission from the pristine tubes, highlighting the key role of the thiol-induced immobilization of the AuNPs at the nanotubes sidewalls.

In the second set of control experiment, we show that the other key element towards successful emission enhancement is the preservation of the pristine optoelectronic properties of the tubes. We generate a set of COOH-covalently functionalized carbon nanotubes (COOH-SWNTs) by following the standard acid treatment. [29] To produce the carboxylic groups onto the tubes sidewalls, we dissolved 1 g of SWNTs in a mixture of 150 ml of H_2SO_4 and 50 ml of HNO_3,

which was heated up to 60°C and stirred for 45 min. Afterwards, the solution was filtered and washed repeatedly with distilled water to reach a pH value of 7. Subsequently, the sample was dried overnight at 60°C. Figure 38a compares the Raman spectra of pristine (SWNT) and carboxylated (COOH-SWNT) tubes. The increase in the intensity of the D band after the carboxylation of the tubes is a clear indication of the increased defectivity introduced by the covalent approach. The optical properties of the tubes are destroyed by the functionalization. We ensured a mild functionalization to preserve some emission from the functionalized tubes.

To attach the thiol-groups onto the tubes, 100 mg of the COOH-SWNTs were dispersed in thionyl chloride (10 ml) by 30 min sonication. Then they were refluxed for 6 h. The excess of thionyl chloride was evaporated and dry DMF was added to the residual compound. Cystein (100 mg) and triethylamine (0.11 ml) were added to the mixture, which was then stirred for 2 h at 25°C and for 12 h at 70°C, respectively. The mixture was cooled and centrifuged (1100 rpm, 1 hour). The product was dialyzed in the alkali aqueous solution (pH 8) for 2 days and then lyophilized.

The IR spectra of COOH-SWNT and SH-COOH-SWNT, reported in Supplementary Figure 38b, and the results of their elemental analysis, reported in Supplementary Table 7, confirmed the successful functionalization: The appearance of a broad absorbance band in the 3015-3630 cm^{-1} region of the IR spectra of SH-COOH-SWNT as well as the shift of the carbonyl absorbance band from 1726 cm^{-1} for COOH-SWNT to 1596 cm^{-1} for SH-COOH-SWNT indicate conjugation of cysteine molecules to the sidewall of carbon nanotubes. The 10% sulfur content of SH-COOH-SWNT also indicates the attachment of cysteine to SWNTs.

Figure 39 compares the emission of pristine tubes with the one of thiol-terminated carboxylated nanotubes before and after gold attachment. After attaching gold nanoparticles, the nanotube

emission gets quenched. This might be a genuine quenching process due to energy transfer between AuNPs and SWNTs. An additional reduction in intensity may arise from reabsorption and scattering in the solution.

Supplementary Methods

Infrared (IR) spectra were recorded using a JASCO spectrometer. Ultrasonic bath (Model: SONOREX, RK255 HZ) was used to disperse materials in solvents. TGA measurements were recorded by a STA 409 apparatus (from Netzsch) in temperatures ranging from 25-800ºC with a 10ºC/min heating rate under air. Elemental analysis was performed using ELEMENTAR apparatus with three columns and detector for carbon, nitrogen, hydrogen and sulfur elements.

XPS measurements were carried out on SWNT, SWNT-low, SWNT-high, and SP-SWNT samples. For this the substances were dispersed in THF and the suspensions carefully dropped onto thin-film gold substrates (300 nm Au(111) on mica, Georg Albert PVD) and left on the bench until the solvent was evaporated. Samples were then brought into ultra-high vacuum (UHV) and measured with a monochromatic high-resolution XPS setup (VG Scienta MX 650 and SES-200) in perpendicular take-off geometry with constant analyzer pass energy of 200 eV. All binding energies (E_B) were referenced to the Au substrate $4f_{7/2}$ peak at E_B = 83.96 eV and are correct within 40 meV. The zero of the binding energy scale corresponds to the Fermi level of the Au substrate. Due to the drop-coating preparation the samples did not contain equal absolute amounts of substance and their spectra needed to be normalized. Considering the comparable shape of the C 1s spectra and the fact that the SWNTs were not exposed to any harsh treatments, we assumed that their carbon backbones remained intact and that the established functionalization ratios (FR) did not significantly reduce the amount of sp^2-hybridized carbon atoms. Therefore we chose to normalize all SWNT XP spectra with respect to the carbon backbone, i.e., the intensity of the sp^2-carbon component.

High resolution TEM was performed employing an imaging-side aberration-corrected FEI Titan-Cube microscope working at 80 kV, equipped with a Cs corrector (CETCOR from CEOS

GmbH). Spatially-resolved electron energy loss spectroscopy (EELS) measurements were performed on probe-corrected scanning TEM (STEM) FEI Titan Low-Base operating at 80keV (fitted with a X-FEG® gun and Cs-probe corrector (CESCOR from CEOS GmbH)). Furthermore, in order to avoid the effects of electron beam damage, these measurements have been performed using a liquid-nitrogen-cooled cryo-holder at -170° C. EEL spectra were recorded using the spectrum-imaging (SPIM in 2D or spectrum-line (SPLI) in 1D) mode [15,30] in a Gatan GIF Tridiem ESR 865 spectrometer. The convergent semi-angle was of 25 mrad, the collection semi-angle was of 30 mrad and the energy resolution ~ 1.0 eV. To filter the noise in the experimental data, the background corrected EEL spectra showed in Supplementary Figure 14f were smoothed using a Savitzky-Golay filter (second-order polynomial).

The transmission electron microscopy (TEM) samples were prepared by dispersing the NTs powders in ethanol. The dispersions were ultrasonicated and subsequently deposited on holey carbon 3 mm copper grids.

Raman spectra of metallic nanotubes were acquired using an XploRa spectrometer (Horiba), excitation wavelength at 532 nm, equipped with charge-coupled device, 2400 lines/mm gratings and edge filter to block Rayleigh-scattered light. Frequencies were calibrated using a cyclohexane reference sample.

The electrospray ionization-time of flight (ESI-TOF) measurements were performed on an Agilent 6210 ESI-TOF from Agilent Technologies, Santa Clara, CA, USA. The solvent flow rate was adjusted to 4 µL/min and the spray voltage set to 4 kV. The drying gas flow rate was set to 15 psi (1 bar). All the other parameters were adjusted for a maximum abundance of the relative $[M+H]^+$. ^1H NMR and ^{13}C NMR spectra were recorded on a Bruker Avance 400 spectrometer

(Bruker Corporation, Billerica, MA, USA) (at 295 K). Tetramethylsilane was used for internal calibration at 125 MHz with complete proton decoupling.

Supplementary References

1. Mori K, Matsuno Y, Mori K, Kudo T. *Bonding method, bondability improving agent, surface modification method, surface modifying agent, and novel compound.* US patent 2013, US20130177770 A1.
2. Bucher G, Siegler F, Jens Wolff J. *Photochemistry of 2-azido-4,6-dichloro-s-triazine: matrix isolation of a strained cyclic carbodiimide containing four nitrogen atoms in a seven-membered ring*. Chemical Communications **20,** *2113-2114* (1999).
3. Gebhardt B, Syrgiannis Z, Backes C, Graupner R, Hauke F, Hirsch A. *Selective Polycarboxylation of Semiconducting Single-Walled Carbon Nanotubes by Reductive Sidewall Functionalization.* J. Am. Chem. Soc. **133**, *7985-7995* (2011).
4. Gao C, He H, Zhou L, Zheng X, Zhang Y. *Scalable Functional Group Engineering of Carbon Nanotubes by Improved One-Step Nitrene Chemistry*. Chem. Mater. **21**, *360-370* (2009).
5. Yadav S. K, Kim J, Kim H. J, Kim J, Hong S. M, Koo C. M. *PDMS/MWCNT nanocomposite actuators using silicone functionalized multiwalled carbon nanotubes via nitrene chemistry*. J. Mat. Chem. C **1**, *5463-5470* (2013).
6. H. Namazi and M. Adeli. *Synthesis of barbell-like triblock copolymers, dendritic triazine-block-poly(ethylene glycol)-block-dendritic triazine and investigation of their solution behaviors,* Polymer **46**, *10788-10799* (2005).
7. J. Maultzsch, H. Telg, S. Reich, and C. Thomsen, *Radial breathing mode of single-walled carbon nanotubes: Optical transition energies and chiral-index assignment,* Phys. Rev. B **72**, *205438* (2005).

8. P. Ayala, R. Arenal, M. Rummeli, A. Rubio, T. Pichler, *Doping carbon nanotubes with Nitrogen: a route towards applications*, Carbon **48**, *575-586* (2010).

9. P. Ayala, R. Arenal, A. Loiseau, A. Rubio, T. Pichler, *The physical and chemical properties of heteronanotubes*, Rev. Mod. Phys. **82**, *1843-1885* (2010).

10. R. Arenal, K. March, C.P. Ewels, X. Rocquefelte, M. Kociak, A. Loiseau, O. Stephan, *Atomic Configuration of Nitrogen-Doped Single-Walled Carbon Nanotubes*, Nano Lett. **14**, *5509−5516* (2014).

11. R. Arenal, X. Blase, A. Loiseau, *Boron-nitride and boron-carbonitride (BCN) NTs: synthesis, characterization and theory*, Adv. in Phys. **59**, *101-179* (2010).

12. L. Alvarez, Y. Almadori, R. Arenal, R. Babaa, T. Michel, R. Leparc, J-L.Bantignies, P. Hermet, J-L.Sauvajol, *Transfer evidence between carbon nanotubes and encapsulated conjugated oligomers,* J. Physical Chem. C **115**, *11898-11905* (2011).

13. R. Arenal, L. De Matteis, L. Custardoy, A. Mayoral, M. Tence, V. Grazu, J.M. de la Fuente, C. Marquina, M.R. Ibarra, *Spatially-Resolved EELS Analysis of the Antibody Distribution on Bio-functionalized Magnetic Nanoparticles*, ACS Nano **7**, *4006-4013* (2013).

14. R. Arenal, F. de la Pena, O. Stephan, M. Walls, A. Loiseau, C. Colliex, *Extending the analysis of EELS spectrum-imaging data, from elemental to bond mapping in complex nanostructures*, Ultramicroscopy **109**, *32-38* (2008).

15. A. Mayoral and R. Arenal, *Advanced Transmission Electron Microscopy: Applications to Nanomaterials,* Eds. L. Francis, Springer (2015).

16. S. Reich, C. Thomsen, J. Maultzsch, *Carbon Nanotubes: Basic Concepts and Physical Properties,* Wiley-VCH (2004).

17. S. R. Bahn and K. W. Jacobsen, *An object-oriented scripting interface to a legacy electronic structure code,* Comput. Sci. Eng. **4,** *56-66* (2002).

18. H.L. Spell, Infrared spectra of N-substituted aziridine compounds. *Anal. Chem.*, **39**, *185–193* (1967).

19. M. Schmid, H.-P. Steinrück, J.M. Gottfried, *A new asymmetric Pseudo-Voigt function for more efficient fitting of XPS lines,* Surf. Interface Anal. **46,** *505–511* (2014).

20. M. Filippi, L. Calliari, *Measuring the energy of the graphite π + σ plasmon peak*, Surf. Interface Anal. **38**, *595–598* (2006).

21. S. Tougaard, *Quantitative analysis of the inelastic background in surface electron spectroscopy,* Surf. Interface Anal. **11**, *453–472* (1988).

22. C.D. Wagner, L.E. Davis, M.V. Zeller, J.A. Taylor, R.H. Raymond, L.H. Gale, *Empirical atomic sensitivity factors for quantitative analysis by electron spectroscopy for chemical analysis,* Surf. Interface Anal. **3,** *211–225* (1981).

23. D.A. Shirley, *High-Resolution X-Ray Photoemission Spectrum of the Valence Bands of Gold,* Phys. Rev. B. **5**, *4709–4714* (1972).

24. J. Allouche, A. Le Beulze, J.-C. Dupin, J.-B. Ledeuil, S. Blanc, D. Gonbeau, *Hybrid spiropyran–silica nanoparticles with a core-shell structure: sol–gel synthesis and photochromic properties*, J. Mater. Chem. **20**, *9370-9378* (2010).

25. J. Cervenka *et al. Graphene field effect transistor as a probe of electronic structure and charge transfer at organic molecule–graphene interfaces,* Nanoscale **7**, *1471* (2015).

26. B. Hatting, S. Heeg, K. Ataka, J. Heberle, F. Hennrich, M.M. Kappes, R. Krupke, and S. Reich, *Fermi energy shift in deposited metallic nanotubes: A Raman scattering study*, Physical Review B **87**, *165442* (2013).

27. M. Lazzeri, S. Piscanec, F. Mauri, A.C. Ferrari, and J. Robertson, *Phonon linewidths and electron-phonon coupling in graphite and nanotubes*, Physical Review B **73**, *155426* (2006).

28. Das, A. Sood, A. K. Govindaraj, A. Marco Saitta, A. Lazzeri, M. Mauri, F. and Rao, C.N.R *Doping in carbon nanotubes probed by Raman and transport measurements.* Phys. Rev. Lett. **99**, 136803 (2007).

29. R. Yu *et al.*, *Platinum Deposition on Carbon Nanotubes via Chemical Modification*, Chem. Mater.**10**, *718-722* (1998).

30. C. Jeanguillaume, C. Colliex, *Spectrum-image: the next step in EELS digital acquisition*, Ultramicroscopy **28**, *252-257* (1989).
